# Derivatization-Assisted Lipase-Mediated Separation of Optically Pure α-Cyclopentylmandelic Acid Through Enhanced Substrate Recognition

**DOI:** 10.3390/molecules31183290

**Published:** 2026-09-17

**Authors:** Yuhao Zhao, Zhizhi Li, Xianwei Long, Qun Lu

**Affiliations:** School of Life Science and Engineering, Southwest Jiaotong University, Chengdu 610031, China

**Keywords:** chiral resolution, lipase, immobilized enzyme, α-cyclopentylmandelic acid, sofpironium bromide

## Abstract

α-Cyclopentylmandelic acid (CPMA) is a chiral α-hydroxycarboxylic acid valuable in pharmaceutical synthesis and asymmetric catalysis. However, it remains challenging to prepare with high optical purity due to its unique steric structure. This study established a chiral resolution strategy using derivatization-assisted lipase-catalyzed kinetic resolution to efficiently prepare optically pure CPMA. We employed acetoxyacetyl chloride derivatization to introduce a diester structure into CPMA that lipase recognizes efficiently and identified recombinant Cal B as the optimal catalyst through enzyme screening. Theoretical calculations suggested that the activity difference resulting from the derivatization strategy stems from spatial recognition rather than electronic effects; the significant difference in binding affinity between the R- and S-substrates provides the driving force for subsequent time-dependent separation. By controlling the reaction time, the (R)- and (S)-CPMA enantiomers could be selectively prepared. This achieved ee values exceeding 99.5% for both enantiomers, with isolated yields over 38% for each. Cal B was immobilized on an ESR carrier and retained good catalytic activity after 12 reuse cycles. The resulting (R)-CPMA was successfully used to synthesize sofpironium bromide, thereby validating the practical feasibility of this process. This strategy provides an effective, environmentally friendly enzymatic route to highly sterically hindered chiral α-hydroxycarboxylic acids.

## 1. Introduction

Chiral α-hydroxycarboxylic acids are an important class of chiral building units widely found in anticholinergic drugs, such as anisodine and oxybutynin [1,2]. They are also important components for chiral ligands and asymmetric catalytic systems, with significant uses in asymmetric synthesis [3,4]. CPMA is a representative chiral α-hydroxycarboxylic acid. Introducing a cyclopentyl substituent at the α-position of mandelic acid endows CPMA with both an aryl hydroxyacid backbone and a hydrophobic cyclic structure, making it highly valuable for chiral construction and related resolution studies [4]. CPMA is a key chiral intermediate in the synthesis of anticholinergic drugs such as sofpironium bromide and glycopyrronium bromide [5,6,7]. The quality of these products depends directly on the optical purity of the key intermediate, (R)-CPMA [6]. There is an urgent need to develop a more environmentally friendly and efficient strategy for the chiral synthesis of CPMA.

However, current methods for preparing optically pure CPMA primarily rely on two strategies: asymmetric synthesis and racemate resolution. Methods based on chiral source synthesis and asymmetric catalysis can avoid traditional resolution processes. Still, they typically require chiral starting materials with high optical purity, and racemization at the chiral center is a risk during key steps such as α-alkylation [6,8]. Alternatively, chromatographic chiral separation strategies based on cyclodextrin-derived chiral selectors have been explored for mandelic acid derivatives and related compounds [9]. However, these approaches generally rely on specialized chiral separation systems and remain challenging for large-scale preparative applications. For chiral resolution, neurotoxic agents such as strychnine and methyl tyrosinate are used as chiral resolving agents [7,10,11], with separation achieved through crystallization of the diastereomeric mixture. These methods generally suffer from high resolving agent consumption, high toxicity, cumbersome crystallization steps, and high solvent requirements. In recent years, enzymatically catalyzed enantioselective conversion strategies have garnered significant attention. Hydrolases, including lipases and proteases, have been widely employed in the chiral resolution of pharmaceuticals and pesticides [12], such as in the preparation of the chiral side chain of paclitaxel [13]. However, due to its unique substrate structure and significant steric hindrance, CPMA may limit effective recognition between the enzyme and the substrate [14]. To address these issues, we conducted a substrate derivatization screening [15]. We developed a derivatization strategy that uses acetyloxyacetylation to construct derivatives efficiently recognized by various lipases. We further screened a recombinant *Candida antarctica* lipase B (Cal B) catalytic system with excellent resolution, thereby enabling efficient preparation of CPMA enantiomers.

Based on the above, this study proposes a new kinetic resolution strategy involving “derivatization-assisted lipase resolution”. By acetyloxyacetylation of CPMA to obtain a substrate suitable for efficient lipase recognition, an optimal catalytic system was determined through enzyme screening and process optimization. Subsequently, immobilization technology was employed to improve the catalyst’s stability and economic viability. Finally, the synthesis of sofpironium bromide was accomplished using the resulting optically pure (R)-CPMA as a starting material, thereby validating the practical feasibility of this process. This study aims to provide a novel route for the green, low-cost preparation of optically pure CPMA and to serve as a reference for the enzymatic resolution of similar chiral α-hydroxycarboxylic acids.

## 2. Results and Discussion

### 2.1. Screening of CPMA Substrate Derivatization Strategies

**Figure 1:**
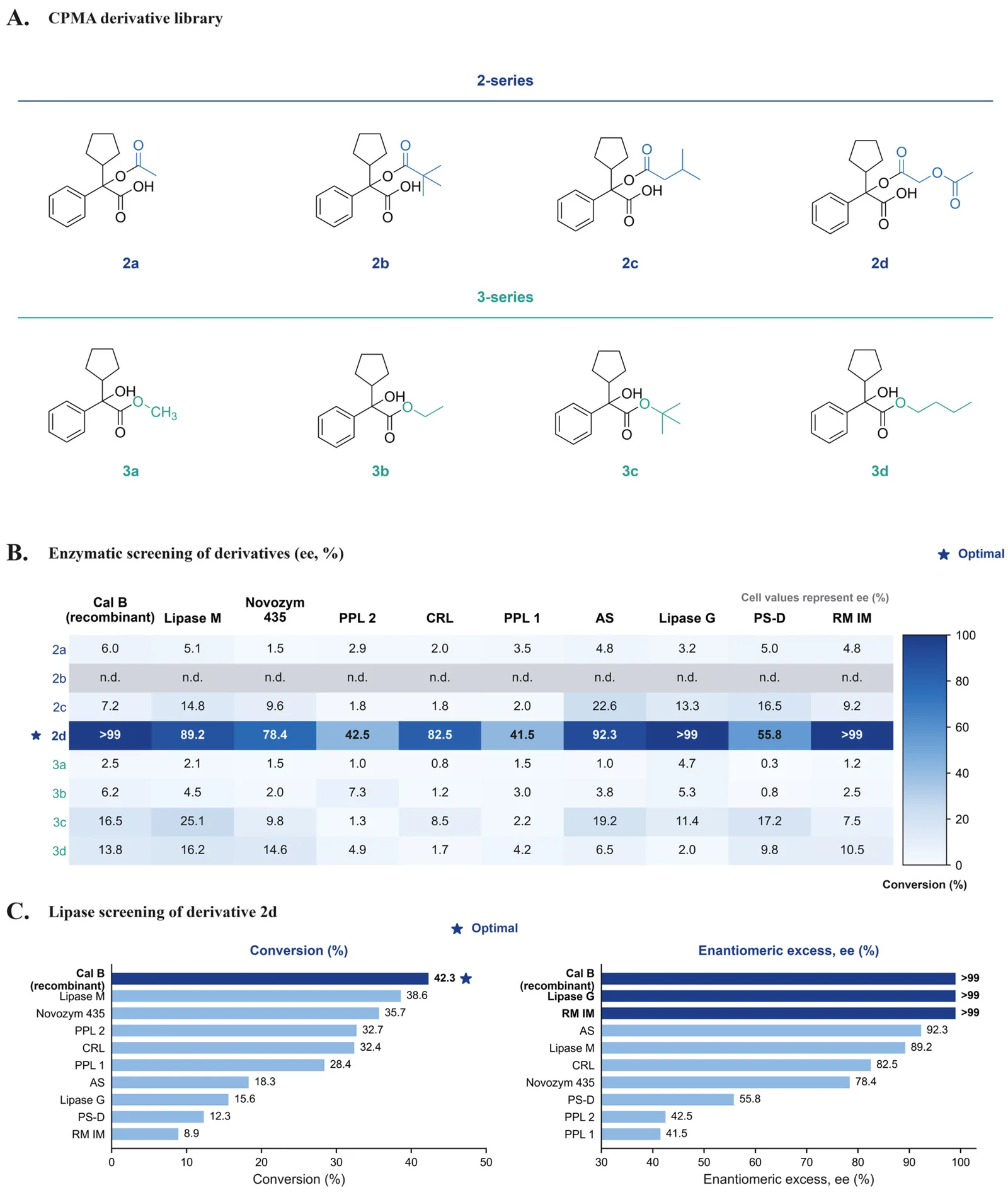
Identification of an optimal CPMA derivative and lipase catalyst through derivatization-assisted screening: (**A**) Chemical structures of CPMA derivatives (**2a**–**2d** and **3a**–**3d**) generated through different derivatization strategies. (**B**) Enzymatic screening of CPMA derivatives based on enantiomeric excess (ee%). (**C**) Lipase screening using derivative **2d** as the substrate based on conversion and enantiomeric excess.

To achieve chiral resolution of CPMA, it is necessary to introduce an ester group that lipases can efficiently recognize. In this study, we first derivatized the substrate and systematically compared two strategies—esterification and acylation—as shown in Figure 1A.

In screening esterification strategies, we used lower-chain fatty alcohols to modify the carboxyl group of CPMA. The resulting monoester derivatives (**3a**–**3b**) exhibited low hydrolytic activity and limited enantiomeric excess (ee < 7%). Further increasing the alkyl chain length or enhancing the steric hindrance of the substituents (**3c**–**3d**), although capable of improving the substrate’s compatibility with lipases to some extent, yielded only limited improvements and failed to achieve highly selective conversions (ee < 25%). These results indicate that simply esterifying the carboxyl group may not be sufficient to confer adequate enzyme recognition capability to the substrate.

Given the limited effectiveness of the esterification strategy, we further evaluated an acylation modification strategy. The tertiary hydroxyl group of CPMA was modified with different acyl substituents. Preliminary evaluation revealed that the tert-butyl-substituted substrate (**2b**) exhibited low hydrolytic activity, whereas the long-chain sec-butyl-substituted substrate (**2c**) demonstrated improved conversion efficiency. However, although porcine pancreatic lipase exhibited some catalytic activity toward certain highly sterically hindered substrates, the products still did not show significant enantioselectivity, suggesting that monoacylation modification may struggle to meet the requirements for both activity and selectivity simultaneously.

Since neither the monoester nor the monoacylation strategies yielded ideal results, we further designed a diester derivatization strategy. Remarkably, substrate **2d**, which incorporates an acetoxyacetyl moiety, achieved highly efficient enantioselective hydrolysis under the catalysis of various lipases. It exhibited high enantioselectivity (ee > 85%) with AS, G, M, RM IM, and Cal B (Figure 1B). Notably, the ee values even exceeded 99% under the catalysis of recombinant Cal B, Lipase G, and Lipozyme RM IM. These results suggest that the outer ester bond in the diester structure may provide a more favorable recognition site for lipases.

Subsequently, we tested the catalytic suitability of different lipases for substrate **2d**. The results showed that recombinant Cal B exhibited the highest catalytic activity and excellent enantioselectivity under mild conditions (Figure 1C). Therefore, this study adopted the **2d**/recombinant Cal B catalytic system as the basis for subsequent process development. Cal B, one of the most widely used commercial lipases, has been extensively applied in biocatalytic processes such as the synthesis of chiral compounds, kinetic resolution, and selective acylation. Its well-established application history and good catalytic stability further support the system’s potential for scale-up [16].

### 2.2. Computational Insights into Derivatization-Assisted Recognition Mechanisms

To elucidate the underlying mechanisms by which the acetyloxy acetylation strategy confers excellent enzymatic catalytic performance on CPMA derivatives and to reveal the source of Cal B’s enantioselectivity toward this substrate, we conducted a multi-level computational study.

To understand the effects of different derivatization reagents on the carbonyl reactivity of chiral alcohol esters, we performed structural optimization and orbital calculations for five substrates, namely **2a**, **2b**, **2c**, **2d**, and CPMA-GA, the glycolic acid ester of CPMA, at the B3LYP-D3(BJ)/6-311G(d,p) theoretical level using the SMD solvent model, as detailed in Figure S1. Frontier molecular orbital analysis revealed that the HOMO–LUMO energy differences among the five substrates were negligible, and the electronic structure of the ester carbonyl group on the chiral alcohol side did not undergo significant changes upon different derivatization. This observation supports the finding that differences in catalytic activity among the derivatization strategies do not stem from changes in the electronic properties of the ester carbonyl itself but instead reflect the influence of the derivatizing groups on spatial recognition between the substrate and the enzyme.

**Figure 2:**
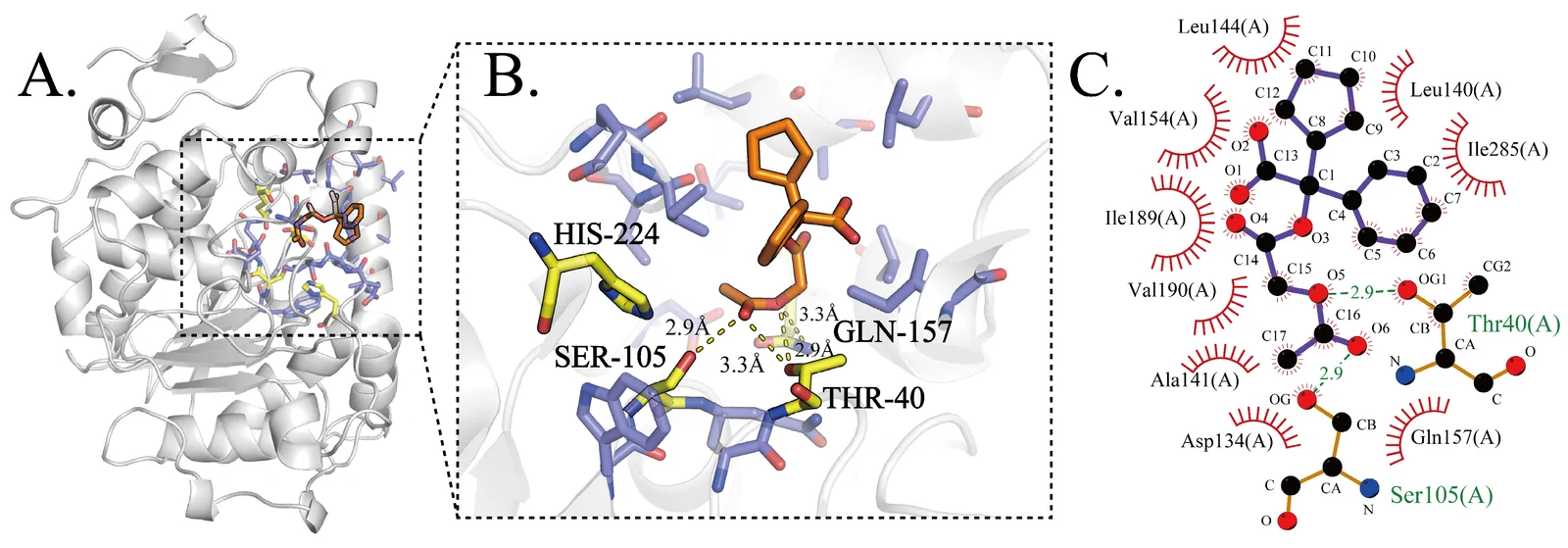
Results of the fully flexible docking of **2d** with Cal B: (**A**) Overall binding mode of substrate (orange) in the active site of Cal B. (**B**) Detailed view of the binding site, with substrate shown in orange sticks, key residues in yellow sticks, other contact residues in blue sticks, and key distances indicated by yellow dashed lines. (**C**) The 2D interaction diagram showing hydrogen bonds and hydrophobic interactions between substrate and active-site residues.

Fully flexible docking further supported this spatial recognition mechanism. As shown in Figure 2, **2d** can stably bind to the active pocket of Cal B. The inset in Figure 2B shows that the distance between the carbonyl oxygen of the outer ester bond and the hydroxyl hydrogen of Ser105 is approximately 2.9 Å, which meets the geometric requirements for nucleophilic catalysis, suggesting that the outer ester bond may be hydrolyzed preferentially. Due to the synergistic steric hindrance imposed by the cyclopentyl group and the benzene ring, if the outer ester bond is not hydrolyzed, the inner ester bond cannot be effectively positioned within the attack range of Ser105. The two-dimensional binding diagram in Figure 2C further corroborates this inference.

**Figure 3:**
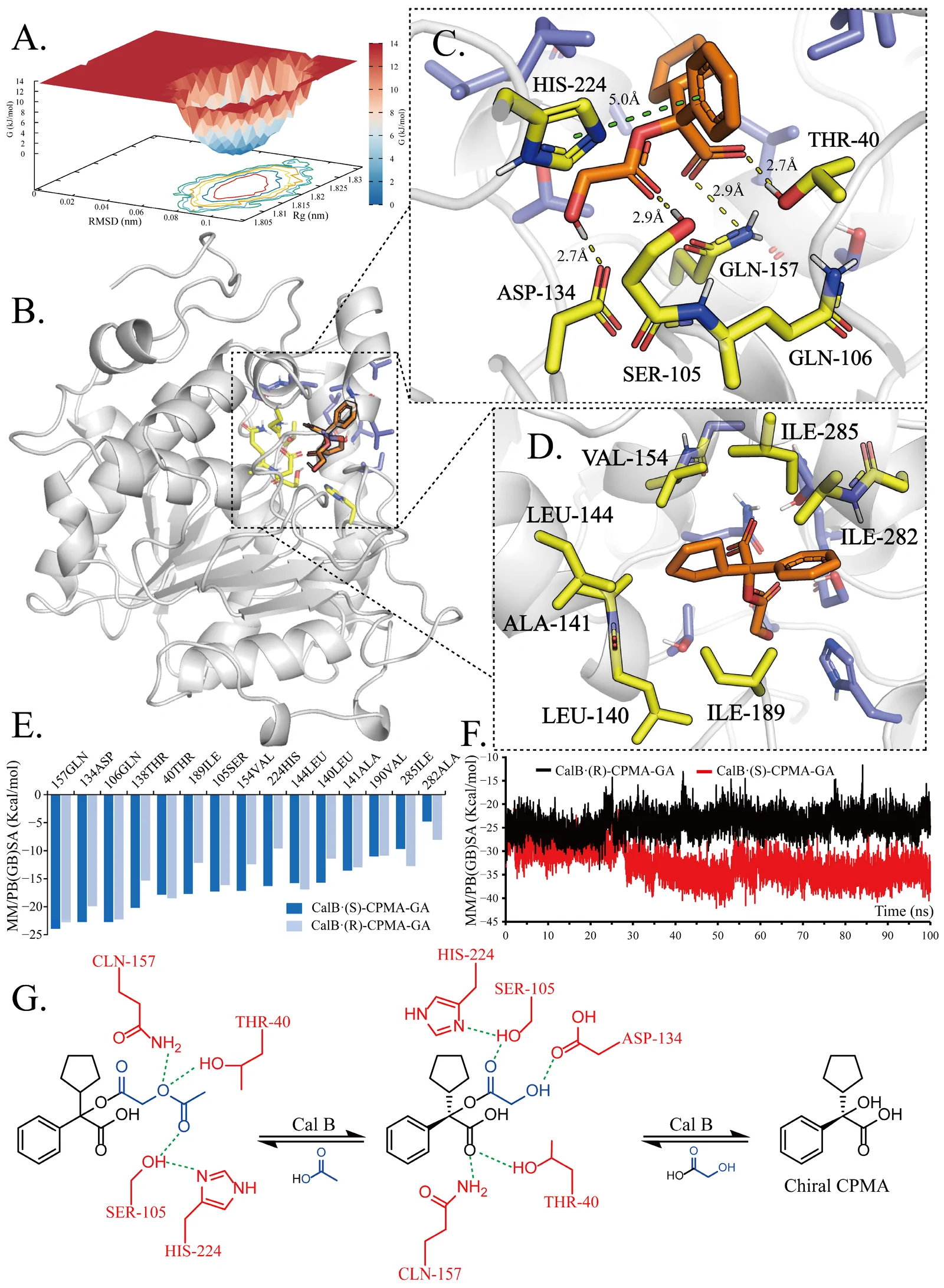
Molecular dynamics simulation results of CPMA-GA with Cal B: (**A**) Free energy landscape (FEL) of the S-substrate–complex system. (**B**) Overall structure of the minimum energy conformation in the potential well, with the substrate shown in sticks. (**C**) Active-site structure and hydrogen-bonding network, with the substrate in orange sticks, key residues in yellow sticks, other contact residues in blue sticks, and key distances indicated by yellow dashed lines. (**D**) Microenvironment of residues involved in localizing the hydrophobic region of the substrate. (**E**) MM-PB(GB)SA residue energy decomposition bar chart. (**F**) Binding free energy trajectories over time for R- and S-substrates. (**G**) Proposed two-step hydrolysis mechanism of CPMA-GA at the Cal B active site. Surrounding catalytic residues are shown in red, while green dashed lines denote potential hydrogen-bonding interactions, and the hydrolyzed moieties are highlighted in blue.

Molecular dynamics simulations of R/S-CPMA-GA with Cal B revealed the origin of stereoselectivity. The free energy landscape (Figure 3A) shows that the simulated system converges to a single potential well. Details of the active site binding (Figure 3C) indicate that Ser105 forms a strong hydrogen bond (2.9 Å) with the carbonyl group of the CPMA-GA glycolate ester bond. Concurrently, Asp134 forms a critical hydrogen bond (2.7 Å) with the hydroxyl group of the glycolate ester bond. This hydrogen bond pulls the substrate toward the interior of the active site, which may be a key factor contributing to the high catalytic activity conferred by this derivatization strategy. Gln154 and Thr40 jointly stabilize the substrate’s carboxyl group, offering a computational rationale for the experimentally observed low activity of carboxyl-derivatized substrates. MM-PB(GB)SA residue decomposition (Figure 3E) shows that the energy contributions from residues providing hydrogen bonds exhibit minimal variation. In contrast, residues involved in localizing the hydrophobic portions of the substrate, specifically Leu140, Ala141, Leu144, Val154, and Ile189, show more pronounced differences. Combined free energy curves (Figure 3F) show that the S-conformation is significantly lower than the R-conformation after 30 ns, with average values of −34.701 kcal/mol and −23.591 kcal/mol, respectively, over the subsequent 20 ns. This provides a quantitative thermodynamic explanation for Cal B’s binding preference for the S-conformation substrate, consistent with the experimentally observed high enantiomeric selectivity (ee > 99.5%). The proposed two-step hydrolysis mechanism is illustrated in Figure 3G, in which the outer ester bond is first preferentially attacked by Ser105, while the hydrogen-bonding network formed by Asp134 and surrounding residues anchors the substrate within the active site to facilitate subsequent cleavage of the inner ester bond. Further analysis of other structural stability metrics, including RMSD, Rg, DCCM, and DSSP, is presented in Figure S2.

**Figure 4:**
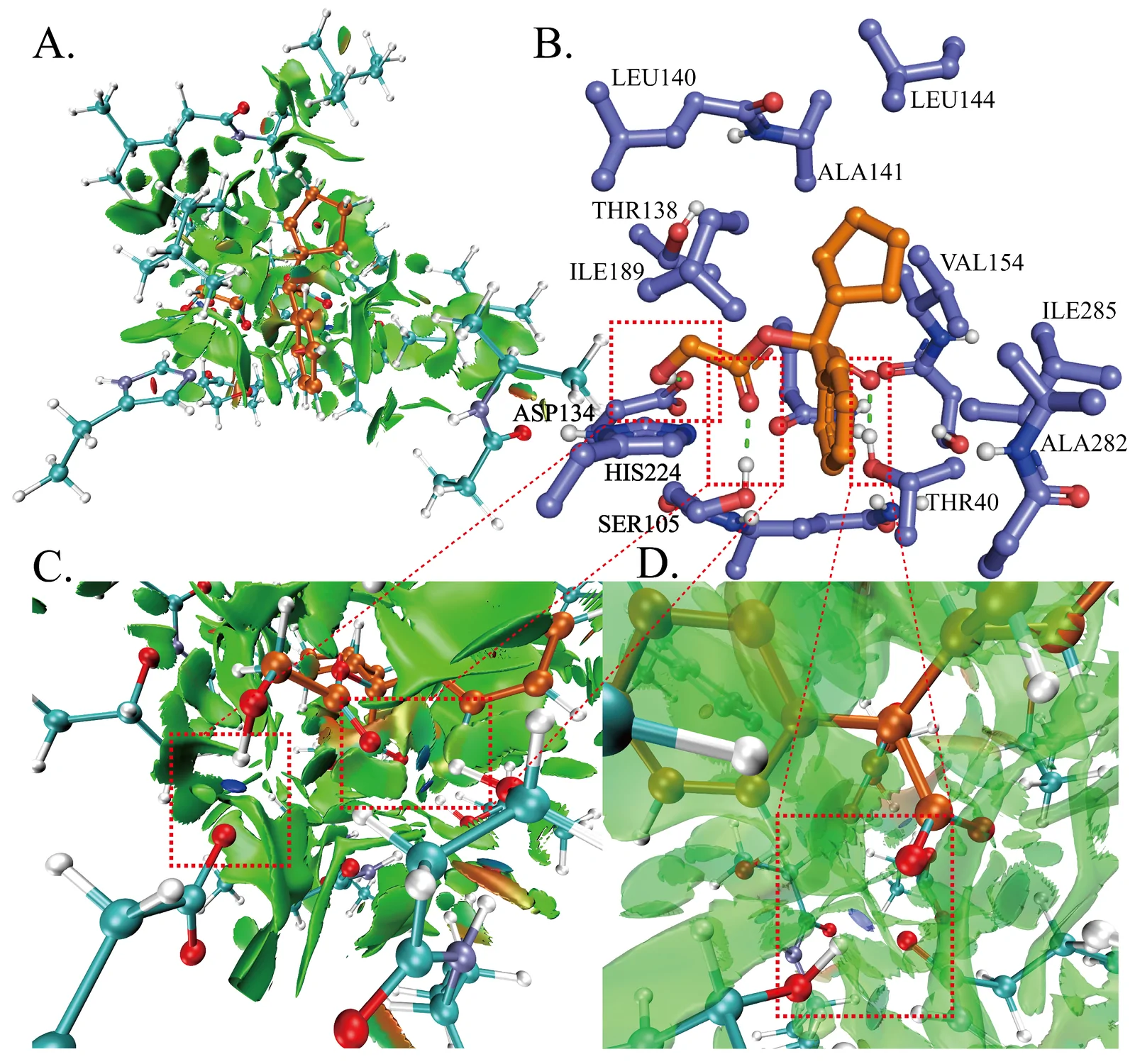
IRI analysis results for the S-form CPMA-GA and the Cal B active site residue cluster: (**A**) Panoramic view of the IRI isosurfaces; the S-form substrate is represented by orange sticks, surrounding residues by light blue sticks, green plate-like isosurfaces represent van der Waals forces, blue isosurfaces represent attractive forces (hydrogen bonds), and red isosurfaces represent repulsive forces. (**B**) Cluster model structure following QM structural optimization; the S-form substrate is represented by orange sticks, surrounding residues by dark blue sticks, and green dashed lines indicate hydrogen bonds. (**C**,**D**) Local IRI enlargements of the corresponding hydrogen-bonding regions, where blue slab-shaped isosurfaces indicate hydrogen-bond interactions.

To gain an intuitive understanding of the binding differences between the R and S enantiomers from an electron-density perspective, we constructed a quantum mechanical cluster model based on the potential-well minimum conformation obtained from molecular dynamics simulations. IRI analysis was performed after structural optimization at the B3LYP-D3(BJ)/6-311G(d,p) level. As shown in Figure 4, in the S-type substrate, the cyclopentyl group is effectively embedded in a hydrophobic cavity formed by residues including Leu140, Ala141, Leu144, Val154, and Ile285, suggesting that this interaction serves as a driving force for stable binding. Meanwhile, the benzene ring of the S-type substrate tends to form a T-type π–π stacking interaction with His224 [17]. Given that His224 abstracts a proton from Ser105 during the catalytic reaction, this stacking may help stabilize its protonated state, thereby favorably influencing the catalytic conversion of the S-type substrate. In contrast, in the R-type substrate, as detailed in Figure S3, the orientation of the cyclopentyl group and the benzene ring is reversed, resulting in a binding mode that differs significantly from that of the S-type substrate. This difference in binding modes between the two enantiomers supports the observed catalytic order, manifested as the preferential conversion of the S-type substrate. The sign(λ_2_)ρ scatter plot, provided in Figure S3, further quantifies this difference.

In summary, we propose a mechanism for the kinetic resolution catalyzed by Cal B with acetyloxy acetylation. The differences in activity among different derivatization strategies are proposed to stem from spatial recognition rather than electronic effects. After preferential hydrolysis of the outer ester bond, the key hydrogen bond between Asp134 and the hydroxyl group pulls the substrate deep into the active site, whereby the anchoring effect of the carboxyl group likely determines the selectivity of the derivatization site. In S-type substrates, the cyclopentyl group is effectively embedded in a hydrophobic cavity, while the benzene ring forms a T-type π–π stacking with His224. This stacking may favorably influence the proton-transfer step in the catalytic cycle, thereby supporting preferential conversion of S-type substrates. The calculated binding free energy is approximately 11.1 kcal/mol lower than that of the R-type substrate, providing a physicochemical basis for the observed high enantioselectivity. This mechanism offers insights into the role of the derivatization strategy across multiple scales, including electronic structure, spatial recognition, hydrogen-bond networks, and weak interactions.

### 2.3. Optimization of Process Conditions for the Cal B Splitting of CPMA

After identifying the substrate and the catalytic enzyme, the Cal B-catalyzed resolution system was further optimized. Drawing on existing process optimization strategies for Cal B-catalyzed systems [18], the effects of reaction temperature, pH, enzyme loading, and reaction time on the catalytic process and the configuration distribution of the products were systematically investigated [19].

**Figure 5:**
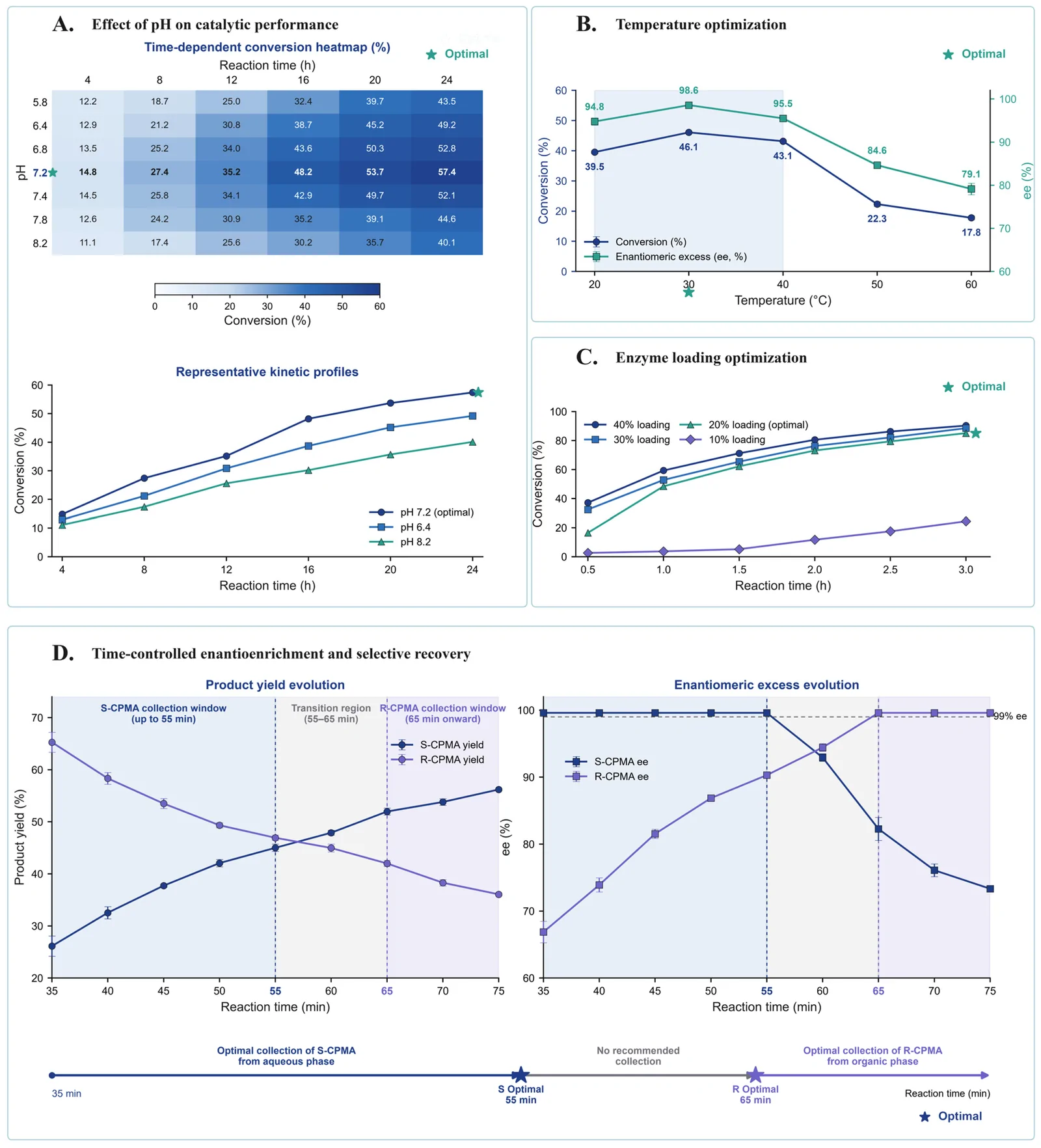
Process optimization and time-controlled selective recovery of CPMA enantiomers during Cal B-catalyzed kinetic resolution: (**A**) Effect of pH on Cal B-catalyzed conversion evaluated by time-dependent reaction profiles. (**B**) Effect of reaction temperature on conversion and enantiomeric selectivity during Cal B-catalyzed resolution. (**C**) Effect of enzyme loading on the catalytic performance of Cal B. (**D**) Time-controlled enantioenrichment and selective recovery of CPMA enantiomers, demonstrating distinct collection windows for S-CPMA and R-CPMA.

The reaction temperature optimization results, shown in Figure 5B, indicate that recombinant Cal B maintains good catalytic activity in the range of 20–40 °C. As the temperature increases, the substrate conversion rate gradually rises, with the highest conversion of 46.1% achieved at 30 °C. However, when the reaction temperature exceeded 40 °C, both the substrate conversion rate and product enantiomeric selectivity decreased significantly. Considering both catalytic efficiency and product enantiomeric selectivity, 30 °C was the optimal reaction temperature for this system.

The effect of reaction pH on the catalytic performance of Cal B is shown in Figure 5A. The conversion of derivative **2d** was evaluated under different pH conditions over a 24 h reaction period. As shown in Figure 5A, Cal B exhibited good catalytic activity within the pH range of 6.4–7.8. The highest conversion was observed at pH 7.2, where conversion increased continuously over time and reached 57.4% after 24 h. In comparison, both lower and higher pH conditions resulted in decreased conversion, indicating that a near-neutral pH was favorable for Cal B-catalyzed hydrolysis. Representative kinetic profiles at selected pH values further showed that the reaction at pH 7.2 proceeded at a higher rate throughout the reaction period. Therefore, pH 7.2 was selected as the optimal condition for subsequent optimization experiments.

The results of the study on the effect of enzyme loading on reaction efficiency (Figure 5C) show that with 20–30% Cal B loading, the reaction rapidly approached the optimal conversion range for kinetic resolution (approximately 50%) within 1 h. When the enzyme loading was reduced to 10%, the initial reaction was significantly slower, and the conversion reached only 24.4% after 3 h. However, once the enzyme concentration exceeded 20%, further increases in enzyme dosage had only a limited effect on conversion, indicating that substrate concentration became a limiting factor. Considering both catalytic efficiency and enzyme cost, we ultimately selected 20% Cal B loading as the optimal condition.

To clarify the selective recovery windows of CPMA enantiomers, we further investigated the effect of reaction time on the kinetic resolution process. Under fixed reaction conditions, we monitored the formation and recovery of S-CPMA and R-CPMA at different reaction times by chiral high-performance liquid chromatography (HPLC) analysis. As shown in Figure 5D, the recovery trends of S-CPMA and R-CPMA changed in opposite directions during the reaction process. In the early stage of the reaction, S-CPMA gradually accumulated and maintained excellent enantiomeric purity, with its ee value remaining above 99% within 35–55 min (Figure 5D). At 55 min, S-CPMA showed high enantiomeric selectivity and favorable recovery, which was considered the optimal collection window for S-CPMA.

With further extension of reaction time, the relative recovery of R-CPMA gradually increased, accompanied by a decrease in S-CPMA recovery. When the reaction time exceeded 65 min, R-CPMA became the major recoverable enantiomer and maintained high optical purity. Based on changes in recovery and ee values, reaction times up to 55 min were considered favorable for S-CPMA collection, whereas reaction times beyond 65 min were suitable for R-CPMA collection. The period between 55 and 65 min was defined as a transition region, where neither enantiomer showed an optimal recovery advantage. These results indicate that reaction time affects not only substrate conversion but also the composition of the recovered CPMA enantiomers, providing a practical approach to selectively preparing S-CPMA and R-CPMA.

### 2.4. Evaluation of Cal B Immobilization and Reusability

Enzyme immobilization technology can enhance catalyst stability and reusability, thereby supporting large-scale biocatalytic processes [20]. To further improve catalyst reusability and reduce enzyme costs [21], this study selected different types of commercial carriers to construct immobilized Cal B and compared their protein adsorption capacity, catalytic performance, and cycle stability (Table 1).

Immobilization systems using different carriers showed significant differences. Carrier properties and their interactions with enzyme molecules influence immobilization efficiency and catalytic performance [22]. The amino-functionalized ESR carrier demonstrated the highest protein adsorption capacity (88.2%), and the immobilized enzyme maintained excellent enantiomeric selectivity (ee > 99.5%). Further cycling experiments showed that the relative activity of Cal B immobilized on ESR remained at 91.7% after 12 cycles, with product ee values consistently above 99.5%. In contrast, the protein adsorption rate on the diatomaceous earth carrier was only 23.3%, and the enantiomeric excess decreased significantly (ee = 77.1%), indicating that the interaction between the carrier and the enzyme has a significant impact on catalytic performance.

The excellent immobilization performance of the ESR carrier can be attributed to the multiple ionic bonds and hydrogen bonds formed between the abundant amino groups on its surface and the carboxyl groups on the surface of Cal B. This noncovalent binding mechanism ensures sufficient adsorption strength while preventing loss of enzyme activity caused by excessive rigidity. Compared with previously reported applications of immobilized Cal B in drug-related biocatalytic processes [23], the stability of the ESR-immobilized system obtained in this study ranks among the highest in similar research. These results indicate that selecting an appropriate immobilization carrier can significantly improve the reusability of Cal B while maintaining its high enantioselectivity; this immobilization system shows great potential for application in the green synthesis of CPMA derivatives.

### 2.5. Preparation of (R)-CPMA and Its Applications in Downstream Synthesis

**Figure 6:**
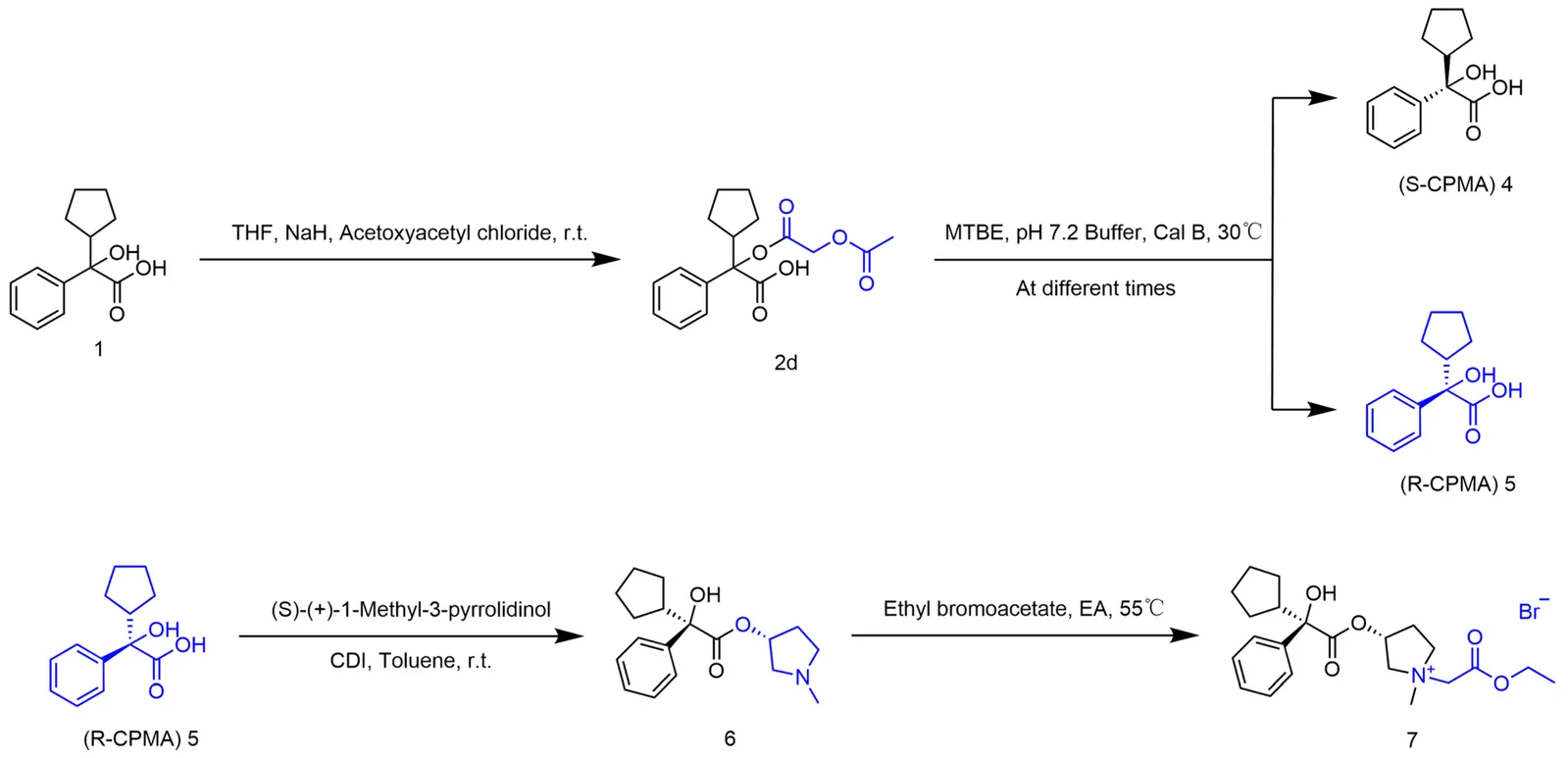
Synthetic validation of optically pure (R)-CPMA through downstream synthesis of sofpironium bromide.

To obtain the target chiral intermediate (R)-CPMA, the separated and enriched (R)-CPMA derivative must undergo deprotection. First, alkaline hydrolysis with sodium hydroxide was attempted; however, even under heated conditions, TLC monitoring showed no significant substrate conversion, suggesting that the derivatization group is highly stable to bases. Subsequently, reflux treatment with 15% hydrochloric acid was employed, which effectively removed the acetyloxyacetyl group. After purification, the white solid (R)-CPMA was obtained. Chiral high-performance liquid chromatography analysis showed that its ee value reached 99.8%; calculated based on racemic CPMA as the starting material, the total isolated yield was 38% (the maximum theoretical yield for kinetic resolution is 50%). These results indicate that the acetyloxyacetyl derivative can undergo successful derivatization-group removal following enzymatic resolution, yielding (R)-CPMA with high optical purity.

(R)-CPMA is a key chiral intermediate in the synthesis of sofpironium bromide; its optical purity directly affects the quality of the final product and serves as a crucial foundation for quality control in subsequent synthetic steps. To further validate the synthetic and application value of the obtained (R)-CPMA, it was used to synthesize sofpironium bromide. (R)-CPMA was condensed with (R)-1-methyl-3-pyrrolidinol in the presence of 1,1′-Carbonyldiimidazole (CDI), followed by quaternization with ethyl bromoacetate to afford sofpironium bromide; the synthetic route is illustrated in Figure 6. The resulting product was identified by ^1^H NMR and HRMS analysis, and its structural data were consistent with those reported in the literature [6], indicating that the (R)-CPMA obtained via the enzyme-catalyzed resolution strategy established in this study can be successfully used in subsequent drug synthesis. This result not only validates the practicality of this process route but also demonstrates that the optical purity of the (R)-CPMA obtained via this strategy fully meets the requirements for drug synthesis.

Furthermore, (S)-CPMA was subjected to the same CDI condensation reaction, and the product was characterized by ^1^H NMR. The full ^1^H NMR spectra of the condensation products obtained from both (R)-CPMA and (S)-CPMA are shown in Supplementary Section S2.

## 3. Materials and Methods

### 3.1. Instruments and Reagents

Instruments included an LC-20AT high-performance liquid chromatograph and SPD-20A detector (Shimadzu, Kyoto, Japan); an AD-RH reverse-chiral column (4.6 mm × 250 mm, 5 μm, Daicel, Osaka, Japan); an INOVA nuclear magnetic resonance spectrometer (TMS internal standard, Varian, Palo Alto, CA, USA); and an LC-MS/MS system (Xevo G2-S TOF, Agilent, Santa Clara, CA, USA). All standard reagents were of analytical grade and commercially available.

Reagents included ESQ, ESR, ES-1, D101 (Tianjin Nankai Hecheng Science & Technology Co., Ltd., Tianjin, China); ReliZyem™ OD-403 (Mitsubishi Chemical Corporation, Tokyo, Japan); diatomaceous earth (Sinopharm Chemical Reagent Co., Ltd., Shanghai, China); recombinant Cal B (constructed and stored in-house); Lipase AS (MedChemExpress, Monmouth Junction, NJ, USA); Lipase G and porcine pancreatic lipase 1 (Shanghai Aladdin Biochemical Technology Co., Ltd., Shanghai, China); Lipase M (Shanghai Yuanye Bio-Technology Co., Ltd., Shanghai, China); Lipase CRL (Sigma-Aldrich, St. Louis, MO, USA); Lipase PS-D (Amano Enzyme Inc., Nagoya, Japan); Novozym 435 and Lipozyme RM IM (Novozymes A/S, Bagsværd, Denmark); Lipase PPL (Shanghai Fusheng Industrial Co., Ltd., Shanghai, China); and Porcine Pancreatic Lipase 2 (Shanghai McLean Biochemical Technology Co., Ltd., Shanghai, China).

### 3.2. Substrate Derivatization and Characterization

NaH (5.0 g, 125 mmol, 60%) was added to a mixture of THF (100 mL) and CPMA (10.0 g, 45.4 mmol) at 0 °C and stirred in an ice bath for 30 min. Acetoxyacetyl chloride (6.9 g, 50.5 mmol) was then added, and the mixture was allowed to warm slowly to rt and stirred for 3 h. The reaction was quenched with 5% HCl (80 mL) and stirred for 20 min. The pH was adjusted to slightly acidic by dropwise addition of 1 M NaOH, and the mixture was stirred for 10 min. The mixture was then extracted with EtOAc, washed with saturated Na_2_CO_3_ solution, and concentrated in vacuo to afford **2d** as a colorless oil (13.5 g, 93% yield). ^1^H NMR (400 MHz, DMSO-d_6_) δ: 1.15~1.29, 1.37~1.49, 1.59~1.76 (m, 8H), 2.18 (s, 3H), 2.80 (tt, *J* = 10.4, 7.4 Hz, 1H), 4.37~4.49 (m, 2H), 7.30~7.44 (m, 5H). HRMS (ESI) *m*/*z*: 319.0968 [M − H]^−^.

### 3.3. Enzyme-Catalyzed Separation and Analytical Methods

To prevent non-enzymatic hydrolysis of the derivatized substrates, all screening experiments were conducted under near-neutral pH conditions. A two-phase reaction system was employed to improve the solubility and dispersion of hydrophobic substrates and facilitate product separation from the reaction system [24]. The derivatized product (20 mg) was dissolved in MTBE (200 μL). A pH 7.4 Tris buffer (1 mL) containing enzyme (5 mg) was prepared. The two phases were mixed and incubated at 37 °C with shaking at 200 rpm. After centrifugation, aliquots were collected from the upper organic and lower aqueous phases to determine substrate conversion and product ee. The following chromatographic conditions were used for the analysis of all screening and optimization experiments (see below for details):

#### 3.3.1. Temperature Screening

The effect of reaction temperature was evaluated at 20, 30, 40, 50, and 60 °C. Each reaction contained substrate **2d** (20 mg), recombinant CalB (5 mg), Tris buffer (1 mL, pH 7.4), and MTBE (200 μL). Reactions were conducted for 40 min at 200 rpm.

#### 3.3.2. pH Screening

The effect of pH was evaluated at seven pH values: 5.8, 6.4, 6.8, 7.2, 7.4, 7.8, and 8.2. Bis-Tris buffer was used at pH 5.8, 6.4, and 6.8. Tris buffer was used at pH 7.2, 7.4, 7.8, and 8.2. All buffer solutions were used at a concentration of 100 mM.

#### 3.3.3. Effect of Enzyme Loading

The amount of recombinant CalB was then optimized: CalB loadings of 1, 2, 3, 4, 5, and 6 mg were evaluated using substrate **2d** (20 mg) under otherwise identical conditions. The reactions were performed in Tris buffer at pH 7.2 and 30 °C for 40 min with shaking at 200 rpm.

Samples were collected at the corresponding reaction times, and the organic phase was directly analyzed by HPLC to determine the residual amount of substrate **2d**. The conversion was calculated accordingly. This sampling and analytical procedure was applied throughout the optimization studies of reaction temperature, pH, and enzyme loading.

#### 3.3.4. Effect of Reaction Time

Finally, the time course of the enzymatic resolution was investigated under the optimized temperature, pH, and enzyme loading conditions. For this experiment, substrate **2d** (200 mg) and recombinant CalB (40 mg) were added to MTBE (2 mL) and Tris buffer (10 mL, pH 7.2). The reaction was conducted at 30 °C with shaking at 200 rpm.

Samples were collected at 5 min intervals from 35 to 80 min. For the reaction time study, both enantiomers were analyzed to monitor the resolution process. After phase separation, the aqueous phase was analyzed directly by chiral HPLC for the unreacted (S)-CPMA. The organic phase was concentrated and treated with 15% HCl under reflux for 1 h to remove the protecting group, followed by extraction and chiral HPLC analysis of the resulting (R)-CPMA.

#### 3.3.5. Chiral HPLC Analysis

The following chromatographic conditions were used for chiral analysis (reversed-phase chiral HPLC): AD-RH chiral column (4.6 mm × 250 mm, 5 μm); mobile phase: water/acetonitrile/formic acid = 600:400:1 (*v*/*v*/*v*), isocratic elution; injection volume: 20 μL; column temperature: 30 °C; flow rate: 0.8 mL·min^−1^; detection wavelength: 220 nm. The retention time for (S)-CPMA was approximately 14.7 min, and that for (R)-CPMA was approximately 18.0 min, as shown in the representative HPLC chromatograms in Supplementary Section S2. The conversion rate was calculated using the following formula:

(1) $$e.e._{p}\%=\frac{\left|S_{R}-S_{S}\right|}{S_{R}+S_{S}}\times 100\%$$

The identities of the (R)- and (S)-CPMA peaks were assigned by comparison with authentic enantiomerically pure reference standards analyzed under identical chromatographic conditions.

### 3.4. Preparation and Performance Evaluation of Immobilized Enzymes

The Bradford assay determined the carrier’s protein adsorption capacity for Cal B. After immobilization, the supernatant was collected, and the free protein concentration was measured using the Coomassie Brilliant Blue G-250 assay. The protein concentration of the enzyme solution without carrier served as the initial protein concentration, and the protein adsorption rate was calculated from the difference in protein concentrations before and after immobilization.

(2) $$\mathrm{Protein}\;\mathrm{adsorption}\;\mathrm{rate}\;(\%)=\frac{C_{0}V_{0}-C_{1}V_{1}}{C_{0}V_{0}}\times 100\%$$

C_0_ and V_0_ represent the protein concentration and volume of the enzyme solution before immobilization, respectively; C_1_ and V_1_ represent the residual protein concentration and volume in the supernatant after immobilization, respectively.

### 3.5. Downstream Synthetic Applications

CPMA (**4** and **5**) Recovery:

The recovery procedure for CPMA enantiomers followed the general procedure described above, and a representative example was performed under the optimized enzymatic resolution conditions using **2d** (200 mg) as the substrate.

(S)-CPMA(**4**): The reaction mixture was centrifuged, and the aqueous phase was collected and adjusted to acidic pH. The aqueous phase was extracted with EtOAc (3×). The organic phases were combined, washed successively with 5% HCl and aq. NaCl, dried over anhydrous Na_2_SO_4_, and then concentrated under reduced pressure to afford S-CPMA (61 mg, 44% yield) as a white solid. ^1^H NMR (600 MHz, CDCl_3_) δ: 1.29~1.77 (m, 8H), 2.96 (p, *J* = 8.3 Hz, 1H), 7.30 (t, *J* = 7.3 Hz, 1H), 7.37 (t, *J* = 7.5 Hz, 2H), 7.68 (d, *J* = 8.1 Hz, 2H). ^13^C NMR (150 MHz, CDCl_3_) δ: 26.03, 26.44, 26.56, 27.09, 47.32, 79.36, 126.05, 127.89, 128.36, 141.05, 180.93. m.p. 119–121 °C (lit. 123–124 °C). [α]^25^_D_ = + 21.8° (c = 1.0, CHCl_3_) [25].

(R)-CPMA(**5**): The reaction mixture was centrifuged, and the organic phase was concentrated under reduced pressure to a small volume. Then, 15% HCl was added, and the mixture was refluxed for 1 h. After completion, the reaction mixture was extracted with EtOAc (3×). The organic phases were combined, washed with aq. NaCl (3×), dried over anhydrous Na_2_SO_4_, and concentrated under reduced pressure to afford R-CPMA (56 mg, 41% yield) as a white solid. ^1^H NMR (600 MHz, CDCl_3_) δ: 1.35~1.76 (m, 8H), 2.96 (p, *J* = 8.3 Hz, 1H), 7.30 (dd, *J* = 12.9, 5.6 Hz, 2H), 7.37 (t, *J* = 7.6 Hz, 2H), 7.68 (d, *J* = 7.4 Hz, 2H). ^13^C NMR (150 MHz, CDCl_3_) δ: 26.03, 26.44, 26.56, 27.09, 47.33, 79.36, 126.00, 127.90, 128.36, 141.05, 180.92. HRMS (ESI) *m*/*z*: 219.0825 [M − H]^−^. m.p. 121–122 °C (lit. 121–122 °C). [α]^25^_D_ = −22.4°(c = 1.0, CHCl_3_, lit. −22.4°) [26].

Synthesis of (3R)-1-methylpyrrolidin-3-yl (2R)-2-cyclopentyl-2-hydroxy-2-phenylacetate (**6**): (R)-CPMA (1 g, 4.5 mmol), CDI (880 mg, 5.5 mmol), and DMF (20 mL) were charged into a flask and stirred in an ice bath for 30 min. 1-Methylpyrrolidin-3-ol (540 mg, 5.5 mmol) was then added dropwise at 0 °C, and the reaction mixture was stirred for 8 h at rt. The reaction was quenched with 5% HCl (100 mL) and stirred for 30 min. The reaction mixture was washed with DCM to remove organic impurities, and the organic layer was discarded. The aqueous layer was basified with solid Na_2_CO_3_, extracted with EtOAc, and the organic layer was concentrated under reduced pressure to afford compound 6 as a yellow oil. ^1^H NMR (400 MHz, CDCl_3_) δ: 1.24~1.47, 1.56~1.69 (m, 8H), 1.84~1.92 (m, 1H), 2.30 (td, *J* = 8.0, 5.9 Hz, 1H), 2.35 (s, 3H), 2.38~2.40 (m, 1H), 2.57 (dd, *J* = 11.1, 2.6 Hz, 1H), 2.69 (dd, *J* = 11.1 Hz, 1H), 2.79 (td, *J* = 8.7, 5.4 Hz, 1H), 2.94 (p, *J* = 8.5 Hz, 1H), 5.21~5.26 (m, 1H), 7.25~7.37, 7.65~7.68 (m, 5H). ESI-MS *m*/*z*: 304.30 [M + H]^+^.

Synthesis of sofpironium bromide (**7**): Compound **6** (1 g, 3.3 mmol) was dissolved in EtOAc (10 mL) at rt. Ethyl bromoacetate (10 mg, 0.06 mmol) was added until a white precipitate formed, then the remaining ethyl bromoacetate (1 g, 5.99 mmol) was added. The reaction mixture was stirred at 55 °C for 1.5 h, then cooled to rt. The solid was collected by filtration, washed with EtOAc, and dried to afford the crude compound as a white solid. The crude solid was refluxed in MTBE/EtOAc for 2 h to afford the quaternary ammonium salt crystals. ^1^H NMR (600 MHz, CDCl_3_) δ: 1.26~1.37, 1.43~1.49, 1.56~1.62, 1.67~1.68 (m, 12H), 2.20~2.24 (m, 1H), 2.86~2.91 (m, 2H), 3.67 (s, 3H), 4.19~4.26 (m, 5H), 4.42 (dd, *J* = 13.9, 5.9 Hz, 1H), 4.68 (q, *J* = 18.2 Hz, 2H), 5.54 (t, *J* = 6.4 Hz, 1H), 7.24~7.28 (m, 1H), 7.35 (q, *J* = 7.5 Hz, 2H), 7.59 (d, *J* = 8.4 Hz, 2H). HRMS (ESI) *m*/*z*: 390.5164 [M − Br]^+^.

### 3.6. Molecular Docking and Molecular Dynamics Simulation

To elucidate the mechanism of derivatization-assisted lipase-catalyzed cleavage at the molecular level, a multi-level theoretical computational study was conducted.

Flexible docking was performed using AutoDock Vina v1.2.3 [27]. The crystal structure of Cal B (PDB ID: 1TCA) [28] was used as the receptor for docking with substrate **2d**. During the docking process, the receptor’s side chains were set to flexible, and all rotatable bonds of the substrate were allowed to rotate freely. The docking box was centered on the catalytic residue Ser105, with dimensions covering the entire active site. Binding modes were evaluated based on a combination of scoring functions and geometric plausibility.

Molecular dynamics simulations were performed using the GROMACS 2020.6 software package [29] with the AMBER14SB force field [30]. Referencing the known crystal structure (PDB: 1TCA), molecular docking was used to construct complex systems of R-CPMA-GA and S-CPMA-GA with Cal B. The systems were placed in a rectangular periodic box with a minimum distance of 1.0 nm, solvated using the TIP3P water model [31,32], and Na^+^ and Cl^−^ ions were added to neutralize the system’s charge. After energy minimization, a 500 ps NVT pre-equilibration followed by a 100 ps NPT pre-equilibration was performed, followed by a 100 ns production simulation under the NPT ensemble. Temperature was controlled using the V-rescale method (303.15 K), and pressure was controlled using the Parrinello–Rahman method (1 bar). MM/PB(GB)SA [33,34] analysis was performed using the gmx_MMPBSA tool [35] in conjunction with the free energy, and a residual energy decomposition was conducted. Free energy topography maps were calculated using the gmx sham module. For details on the analysis of other structural stability metrics (RMSD, Rg, DCCM, DSSP), see Section S2 of the Supplementary Materials.

### 3.7. Quantum Chemical Calculations

All quantum chemical calculations were performed using the Gaussian 16 software package, employing the B3LYP-D3(BJ) functional [36,37,38] and the 6-311G(d,p) basis set [39,40], with the SMD solvent model [41] used to simulate the aqueous environment. The Frontier Molecular Orbital (FMO) analysis covered five substrates: the acetylated product of the tertiary alcohol (**2a**), the pivaloylated product of the tertiary alcohol (**2b**), the tert-alcohol isovaleroyl derivative (**2c**), the tert-alcohol acetyloxy acetyl derivative (**2d**), and the corresponding compound CPMA-GA, obtained by removing the terminal acetyloxy group from **2d**.

The quantum chemical cluster model was constructed based on the lowest-energy conformation identified in the free-energy potential well of molecular dynamics simulations. Centered on the substrate, residues within a 4 Å radius whose atoms fall within a 4 Å spherical shell around the substrate were selected; their α-carbons were truncated and frozen. Structural optimization was performed at the B3LYP-D3(BJ)/6-311G(d,p) theoretical level using the SMD solvent model. Subsequently, the Multiwfn 3.8 [42,43] was used to perform Independent Gradient Model (IRI) analysis [44] to visualize noncovalent interactions.

## 4. Conclusions

This study presents a derivatization-assisted lipase-mediated kinetic resolution strategy that addresses the challenge of preparing optically pure CPMA, a sterically hindered chiral α-hydroxycarboxylic acid. By introducing an acetoxyacetyl diester moiety, the substrate’s spatial profile was reconfigured to enable efficient recognition by recombinant Cal B, achieving ee values exceeding 99.5% for both enantiomers with isolated yields over 38%. The immobilized Cal B on the ESR carrier retained over 91% relative activity after 12 cycles while maintaining enantioselectivity above 99.5%, and the resulting (R)-CPMA was successfully applied in the synthesis of sofpironium bromide, confirming the practical feasibility of this process.

Multilevel computational analyses, including frontier molecular orbital calculations, fully flexible docking, molecular dynamics simulations, and IRI analysis, collectively suggest that the enhanced catalytic performance originates from improved spatial complementarity between the derivatized substrate and the enzyme active pocket, rather than from electronic activation of the ester carbonyl. This mechanistic insight distinguishes our approach from conventional electronic-effect-driven derivatization strategies and highlights the importance of steric matching in lipase–substrate recognition for bulky chiral alcohols.

Despite these advances, several challenges remain that define future research directions. Current substrate engineering approaches, including ours, still rely heavily on empirical screening, and the rational design of derivatizing groups tailored to specific lipase active-site architectures remains underdeveloped. Recent progress in machine learning-guided enzyme–substrate matching and generative models for biocatalyst-compatible substrate design offers promising avenues to accelerate this optimization cycle, as demonstrated in related studies [45]. A previous study has demonstrated that lipase-mediated resolution can be applied to various mandelic acid derivatives, indicating the feasibility of enzymatic transformation of structurally diverse substrates within this class [46]. Beyond improving the design methodology itself, the generality of the diester derivatization paradigm should be examined across other sterically demanding chiral α-hydroxy acids and extended to hydrolases beyond Cal B, thereby broadening the scope of this strategy for green chiral synthesis. Furthermore, whether this derivatization paradigm can be coupled with dynamic kinetic resolution, wherein a racemization catalyst continuously interconverts the enantiomers [47], to surpass the 50% theoretical yield limit of classical kinetic resolution for CPMA and related substrates warrants further investigation.

Most importantly, the transition from batch-mode immobilized biocatalysis to continuous-flow processing represents a critical next step toward industrial implementation. Continuous-flow platforms have demonstrated significant advantages in space-time yield, process safety, and scalability for lipase-catalyzed chiral syntheses, yet their integration with derivatization-assisted kinetic resolution for sterically demanding α-hydroxy acids has not been explored. The robust operational stability of ESR-immobilized Cal B observed herein provides a solid foundation for packed-bed or microreactor configurations where sustained enantioselectivity and continuous product output are prerequisites for economically viable manufacturing. Future efforts should therefore focus on engineering continuous-flow protocols, including optimization of residence time, substrate loading, and in-line deprotection, to enable uninterrupted production of enantiopure CPMA with improved space-time yield and reduced enzyme cost per kilogram of product. Addressing these challenges will further bridge the gap between laboratory-scale enzymatic resolution and industrially viable green manufacturing of high-value chiral pharmaceutical intermediates.

## Figures and Tables

**Table 1:** Effect of support materials on immobilization performance of Cal B ^a^.

Support	Functional Group	Protein Immobilization Efficiency (%)	ee (%)	Relative Activity After 12 Consecutive Cycles ^b^ (%)
ESR	-NH_2_	88.2 ± 0.9	>99.5	91.7
ESQ	-NH_2_	73.8 ± 1.2	>99.5	69.7
ES-1	epoxy	33.5 ± 0.8	>99.5	40.2
OD 403	alkyl	67.4 ± 0.9	>99.5	23.2
Diatomite	Inorganic mineral support	23.3 ± 1.7	77.1	31.4

^a^ Cal B (20 mg) and different supports (200 mg) were incubated in PBS buffer (10 mL, 100 mM, pH 7.0) at 30 °C for 24 h. The immobilized enzymes were obtained by filtration, washing, and drying. ^b^ Substrate (500 mg) was dissolved in MTBE (5 mL), Tris buffer (10 mL, pH 7.0), and immobilized Cal B preparation (1 g) at 30 °C for 70 min. After each cycle, the immobilized enzyme was recovered, washed, and reused for the subsequent cycle.

## Data Availability

The data presented in this study are available in the article and Supplementary Materials.

## Supplementary Materials

The following supporting information can be downloaded at https://www.mdpi.com/article/10.3390/molecules31183290/s1, Section S1: Supplementary Computational Data. Table S1: HOMO and LUMO energies of the five substrates calculated. Figure S1: Frontier molecular orbital analysis of the five substrates. (A–E): HOMO orbitals of **2a**, **2b**, **2c**, **2d**, and CPMA-GA, respectively. (F–J): Corresponding LUMO orbitals. Figure S2: Additional molecular dynamics simulation metrics for R/S-CPMA-GA with Cal B. (A): Free energy landscape of the R-substrate–complex system. (B): RMSD curves of R- and S-substrates during the 100 ns simulation. (C): Radius of gyration (Rg) curves. (D,E): Dynamic cross-correlation matrices (DCCM) for the R- and S-substrate systems, respectively. (F,G): Secondary structure assignment (DSSP) over time for the R- and S-substrate systems, respectively. Figure S3: IRI analysis results for the R-form CPMA-GA and the Cal B active site residue cluster. (A): Panoramic view of the IRI isosurfaces. The R-form substrate is represented by orange sticks, surrounding residues by light blue sticks. Green plate-like isosurfaces represent van der Waals forces, blue isosurfaces represent attractive forces, and red isosurfaces represent repulsive forces. (B): Cluster model structure following QM structural optimization. The R-form substrate is represented by orange sticks, surrounding residues by dark blue sticks, and green dashed lines indicate hydrogen bonds. (C,D): Local IRI enlargements of the corresponding hydrogen-bonding regions, where blue slab-shaped isosurfaces indicate hydrogen-bond interactions. Section S2: ^1^H NMR, ^13^C NMR, MS, and HPLC Spectra of key compounds.
