# Supplementary material for: Derivatization-Assisted Lipase-Mediated Separation of Optically Pure α-Cyclopentylmandelic Acid Through Enhanced Substrate Recognition
Source: Molecules. 2026 Sep 17;31(18):3290. doi: 10.3390/molecules31183290 (PMC13609851; doi:10.3390/molecules31183290)

Supporting Information

Derivatization-Assisted Lipase-Mediated Separation of Optically Pure α-Cyclopentylmandelic Acid Through Enhanced Substrate Recognition

Yuhao Zhao, Zhizhi Li, Xianwei Long and Qun Lu *

School of Life Science and Engineering, Southwest Jiaotong University,

Chengdu 610031, China

*	Correspondence: luqun1125@home.swjtu.edu.cn

Table of Contents

Section S1. Supplementary Computational Data 2-4

Section S2. ^{1}H NMR, ^{13}C NMR, MS, and HPLC Spectra..5-19

S1. Supplementary Computational Data

Table S1. HOMO and LUMO energies of the five substrates calculated.

Substrate	HOMO (eV)	LUMO (eV)
**2a**	-7.0338	-0.6949
**2b**	-6.9598	-0.6551
**2c**	-7.0225	-0.6745
**2d**	-7.1261	-0.8403
**CPMA-GA**	-7.0731	-0.7579

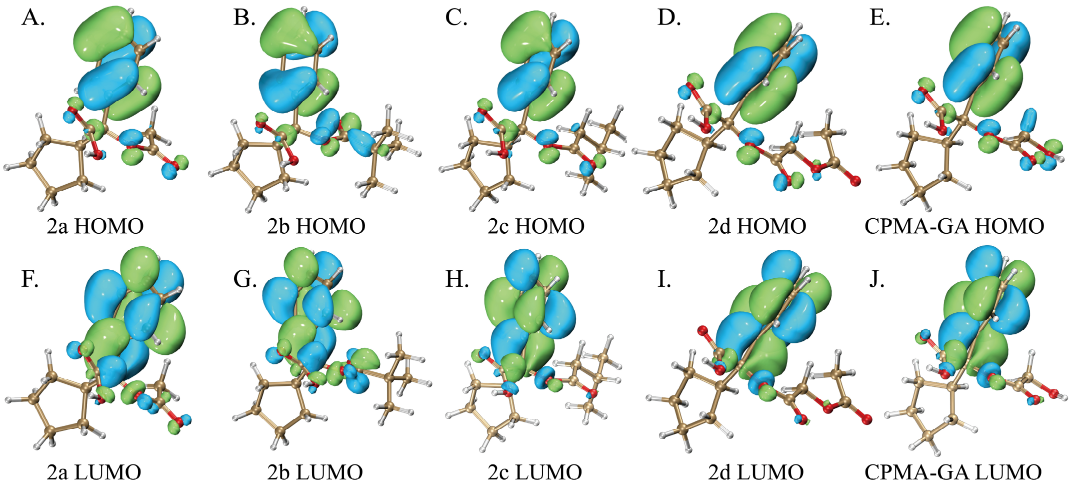

Figure S1. Frontier molecular orbital analysis of the five substrates. (A–E): HOMO orbitals of **2a**, **2b**, **2c**, **2d**, and CPMA-GA, respectively. (F–J): Corresponding LUMO orbitals.

As shown in Table S1 and Figure S1, the HOMO and LUMO energies of the five substrates exhibit minimal variation, with HOMO values ranging from −6.96 to −7.13 eV and LUMO values ranging from −0.66 to −0.84 eV. This observation supports the conclusion that the differences in catalytic activity among the derivatization strategies do not stem from changes in the electronic properties of the ester carbonyl group.

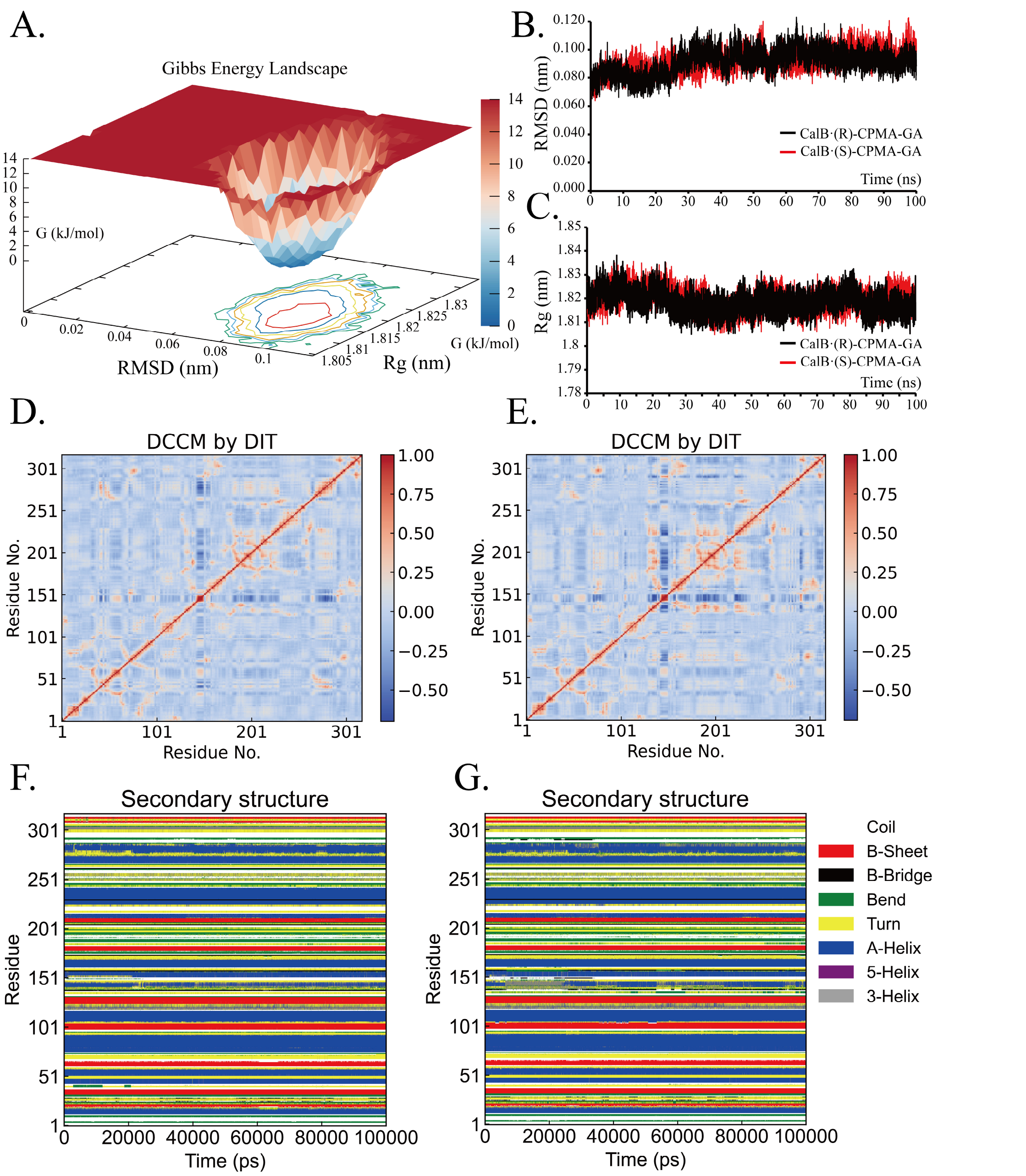

Figure S2. Additional molecular dynamics simulation metrics for R/S-CPMA-GA with Cal B. (A): Free energy landscape of the R-substrate–complex system. (B): RMSD curves of R- and S-substrates during the 100 ns simulation. (C): Radius of gyration (Rg) curves. (D,E): Dynamic cross-correlation matrices (DCCM) for the R- and S-substrate systems, respectively. (F,G): Secondary structure assignment (DSSP) over time for the R- and S-substrate systems, respectively.

As shown in Figure S2, the RMSD and Rg profiles of the R- and S-substrate systems are highly similar throughout the 100 ns simulation, indicating comparable overall structural stability. The DCCM and DSSP analyses further confirm that no significant differences in dynamic behavior or secondary structure evolution were observed between the two systems. These results suggest that the observed enantioselectivity arises from specific binding interactions within the active site rather than from global conformational differences.

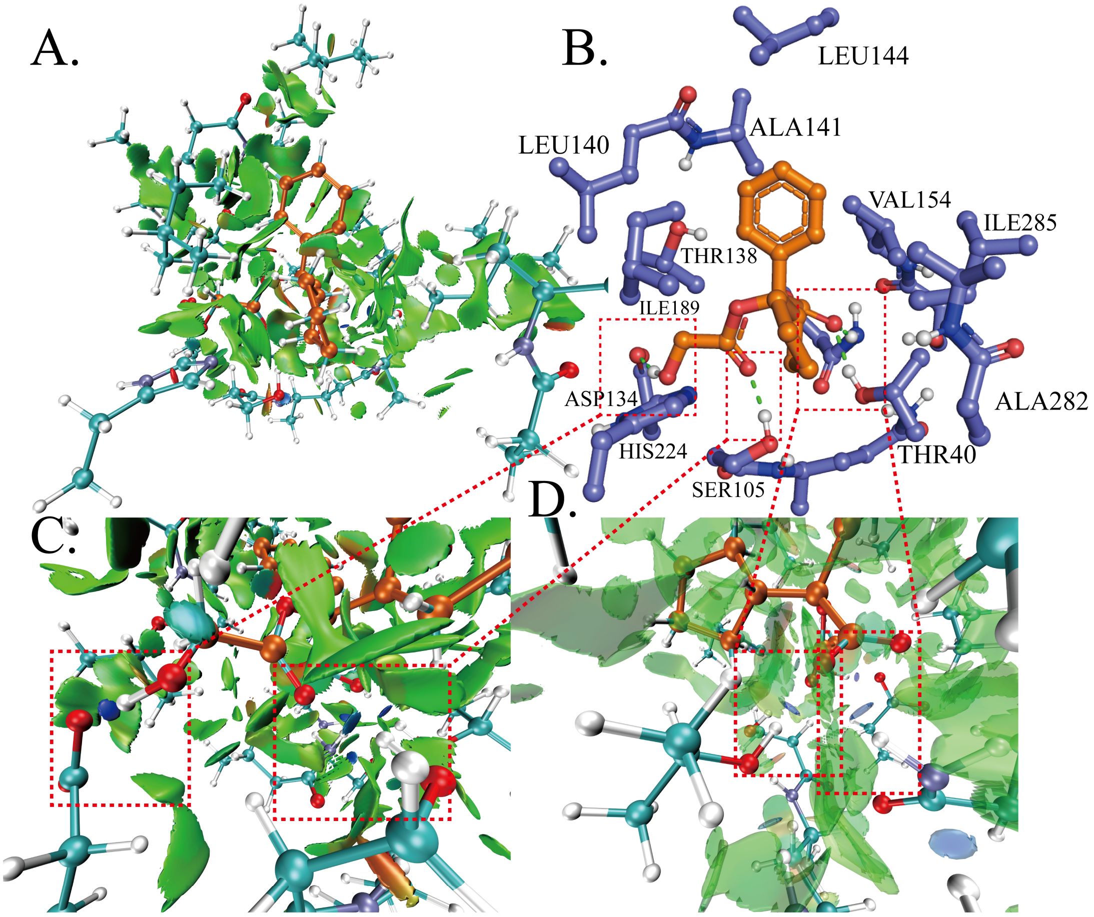

Figure S3. IRI analysis results for the R-form CPMA-GA and the Cal B active site residue cluster. (A): Panoramic view of the IRI isosurfaces. The R-form substrate is represented by orange sticks, surrounding residues by light blue sticks. Green plate-like isosurfaces represent van der Waals forces, blue isosurfaces represent attractive forces, and red isosurfaces represent repulsive forces. (B): Cluster model structure following QM structural optimization. The R-form substrate is represented by orange sticks, surrounding residues by dark blue sticks, and green dashed lines indicate hydrogen bonds. (C,D): Local IRI enlargements of the corresponding hydrogen-bonding regions, where blue slab-shaped isosurfaces indicate hydrogen-bond interactions.

As shown in Figure S3, in the R-type substrate, the orientation of the cyclopentyl group and the benzene ring is reversed compared to the S-type substrate. This reversal leads to a different binding mode within the active site, where the cyclopentyl group is no longer optimally accommodated by the hydrophobic cavity formed by Leu140, Ala141, Leu144, Val154, and Ile285, and the benzene ring cannot adopt a favorable geometry for π-π stacking with His224. This comparative analysis highlights that the primary structural difference between the R- and S-enantiomers lies in the spatial arrangement of these two hydrophobic moieties, which underpins their distinct catalytic behaviors.

S2. ^{1}H NMR, ^{13}C NMR and MS Spectra

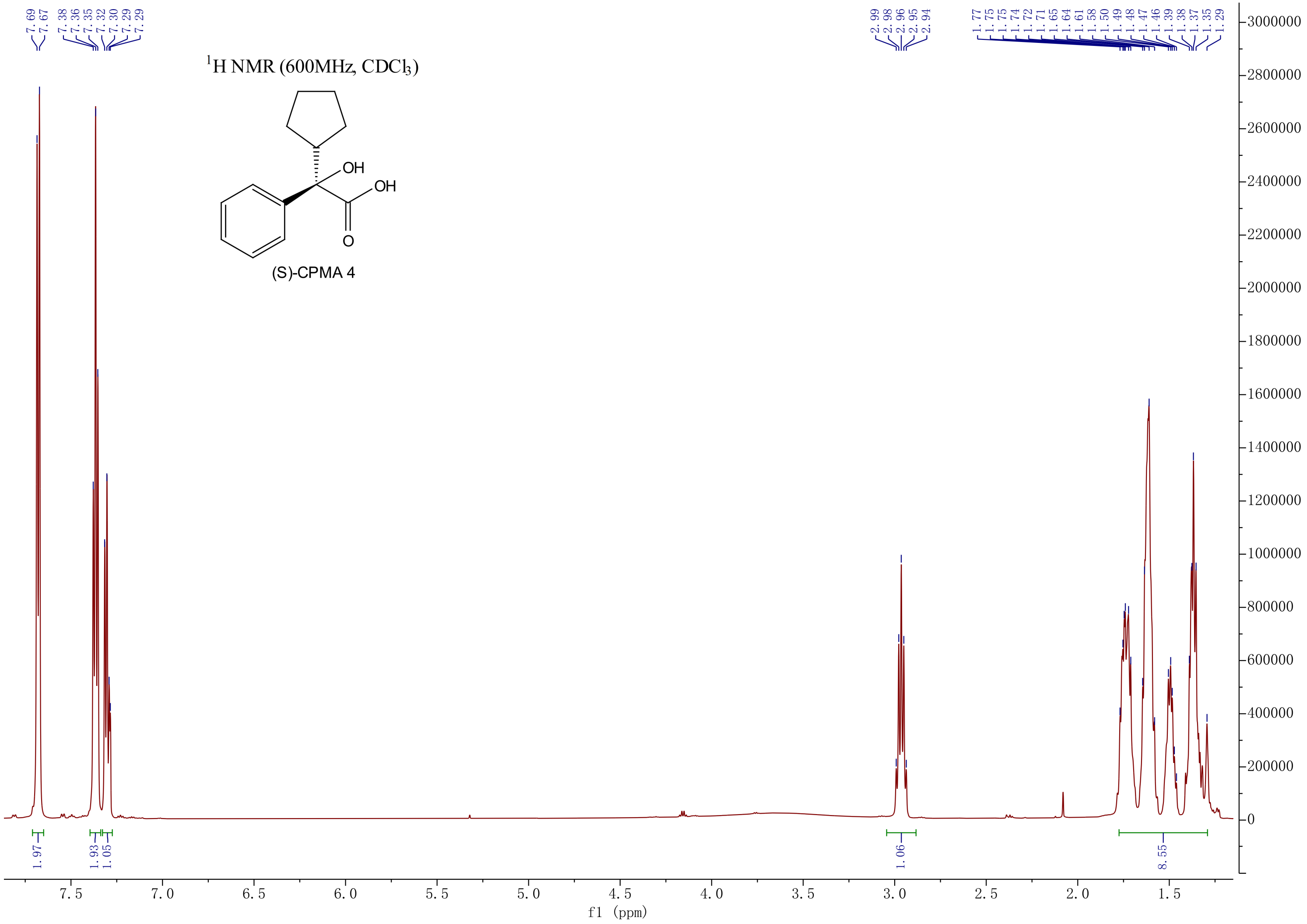

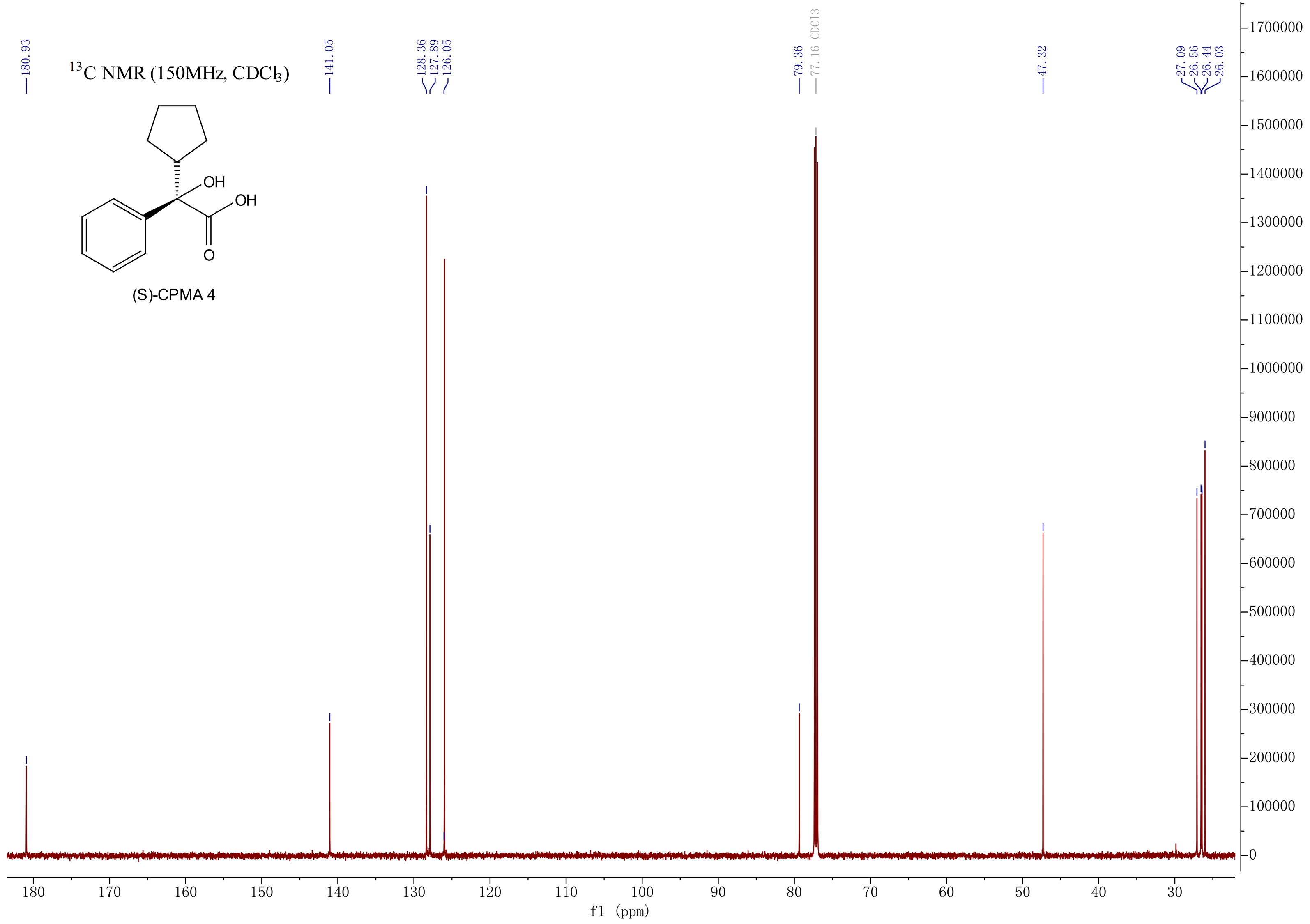

13C NMR (150MHz, CDCl3)
OH
OH
O
(S)-CPMA 4
180.93
141.05
128.36
127.89
126.05
79.36
77.16 CDCl3
47.32
27.09
26.56
26.44
26.03
f1 (ppm)

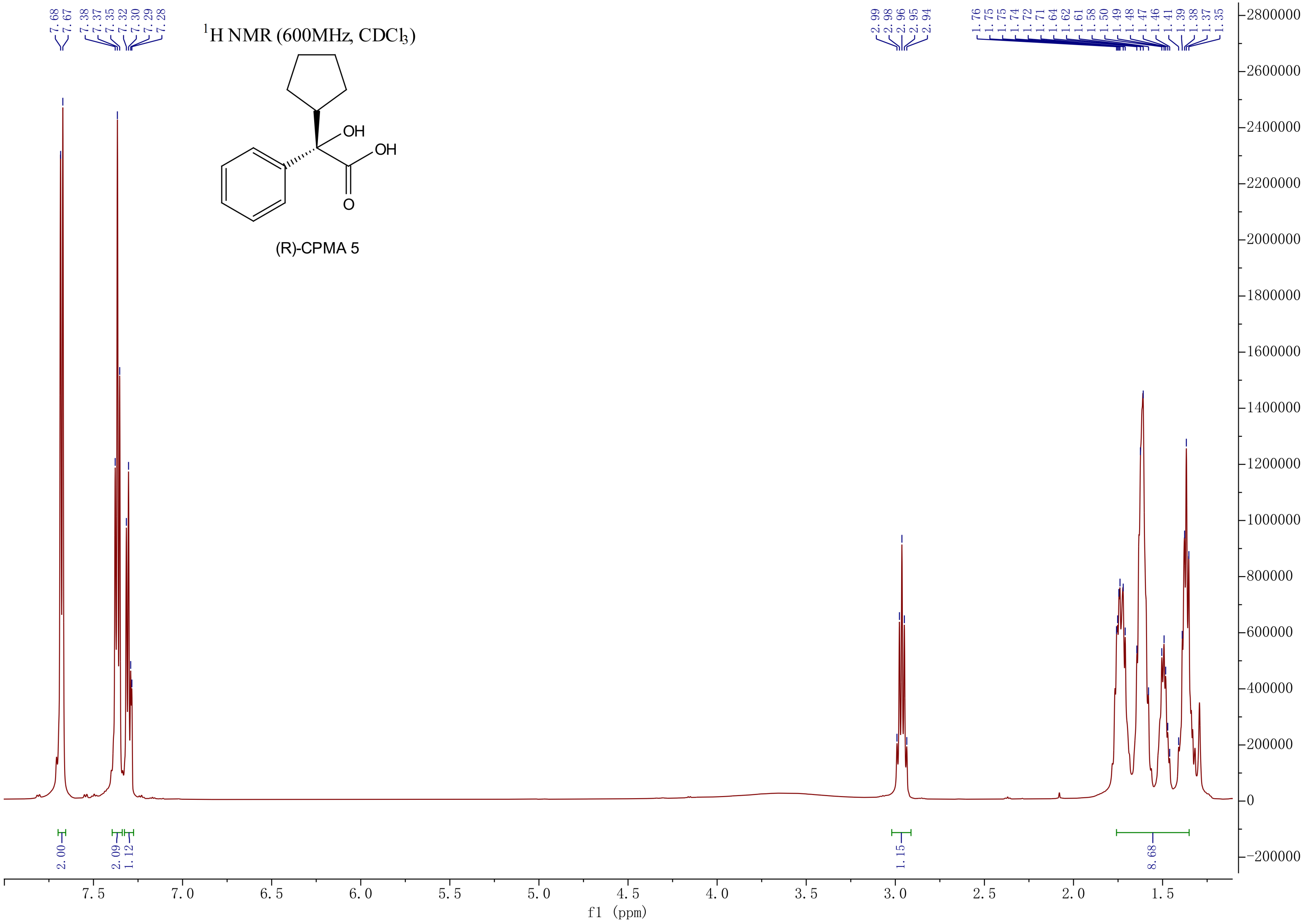

^{1}H NMR (600MHz, CDCl3)
OH
OH
O
(R)-CPMA 5
7.68
7.67
7.38
7.37
7.35
7.32
7.30
7.29
7.28
2.99
2.98
2.96
2.95
2.94
1.76
1.75
1.75
1.74
1.72
1.71
1.64
1.62
1.61
1.58
1.50
1.49
1.48
1.47
1.46
1.41
1.39
1.38
1.37
1.35
2.00
2.09
1.12
1.15
8.68
f1 (ppm)

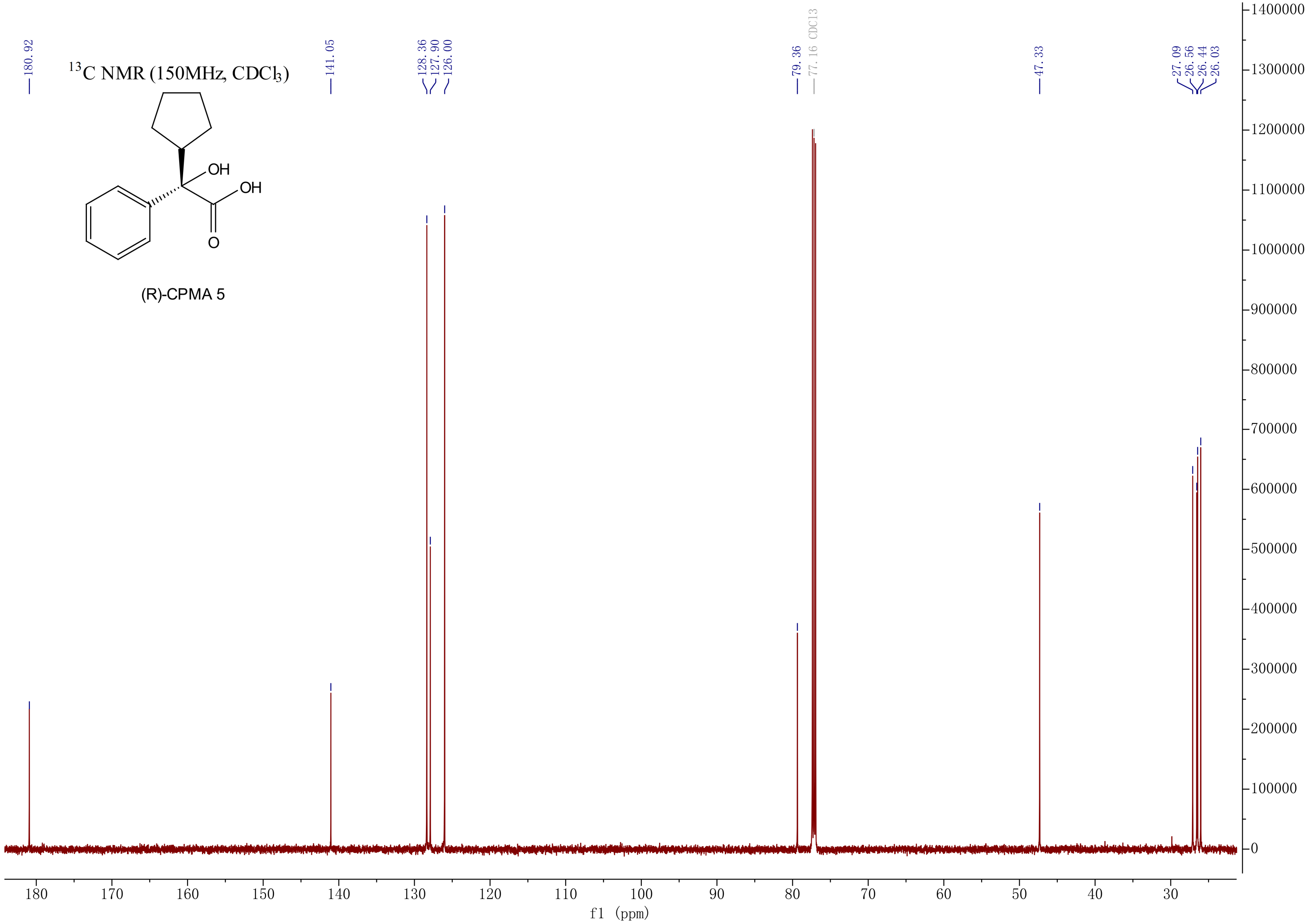

^{13}C NMR (150MHz, CDCl$_3$)
(R)-CPMA 5
OH
OH
O
180.92
141.05
128.36
127.90
126.00
79.36
77.16 CDCl3
47.33
27.09
26.56
26.44
26.03
f1 (ppm)

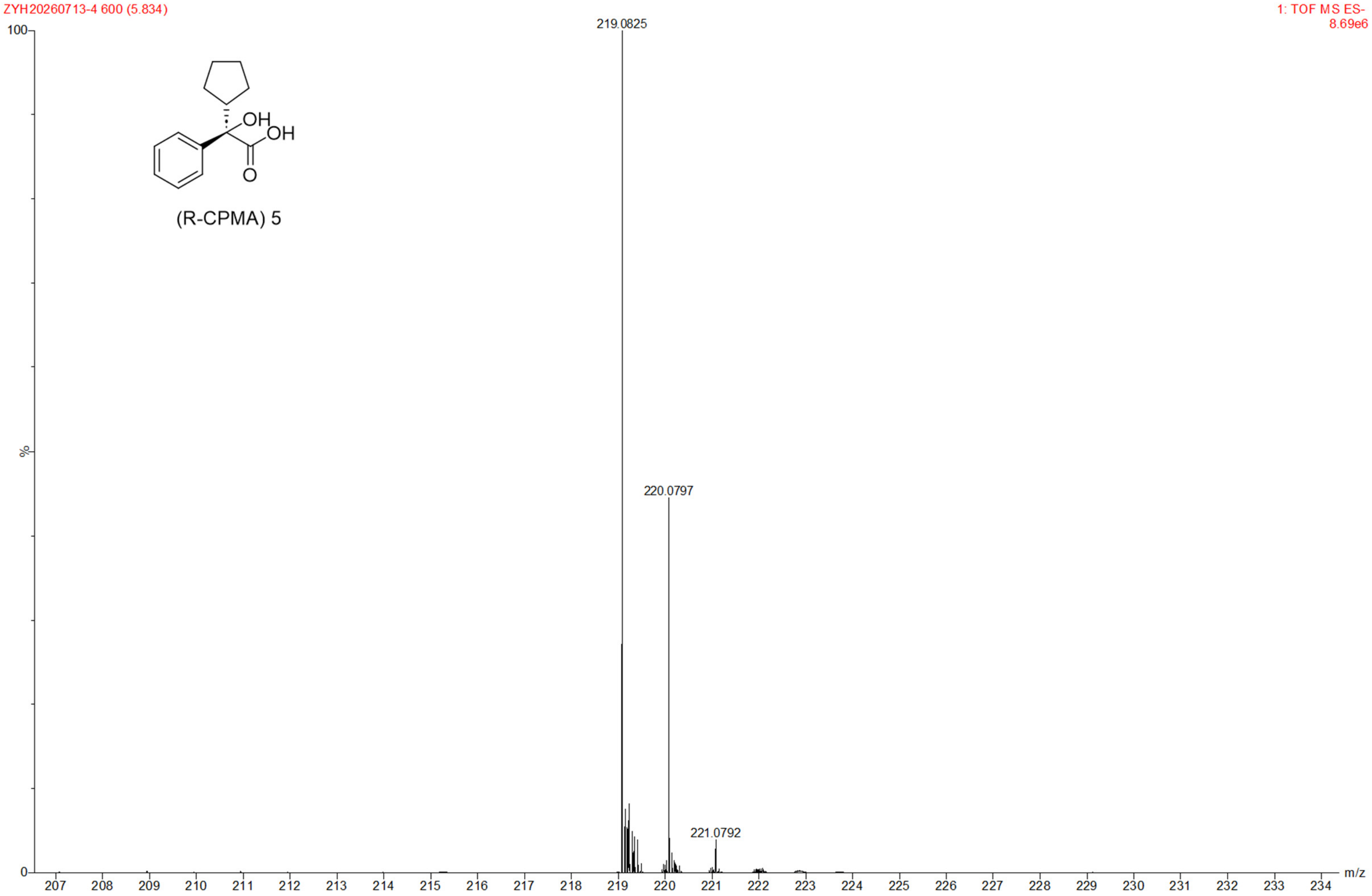
ZYH20260713-4 600 (5.834)
1: TOF MS ES-
8.69e6
219.0825
220.0797
221.0792
OH
OH
O
(R-CPMA) 5
100
%
0
207
208
209
210
211
212
213
214
215
216
217
218
219
220
221
222
223
224
225
226
227
228
229
230
231
232
233
234
m/z

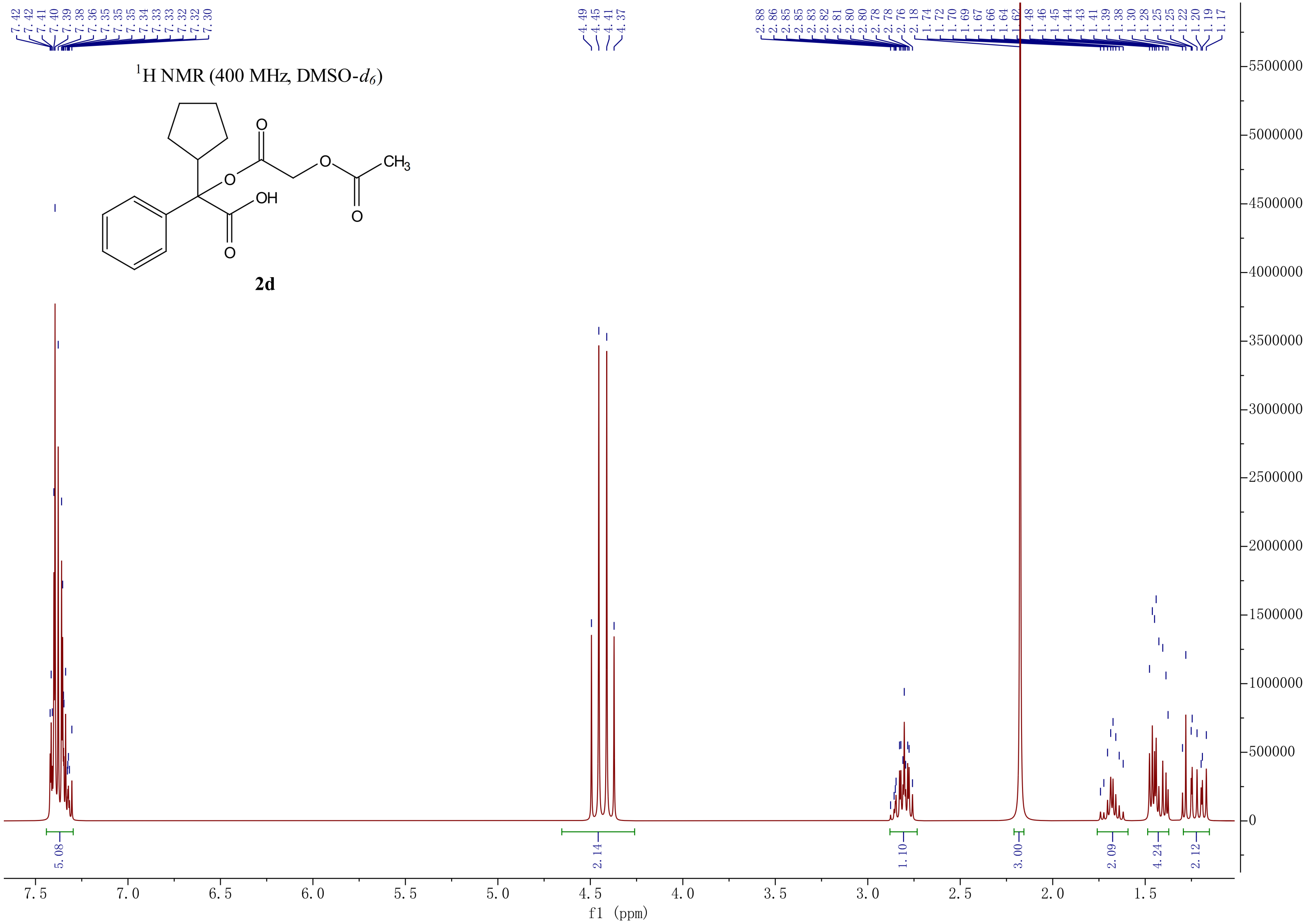
7.42 7.42 7.41 7.40 7.39 7.38 7.36 7.35 7.35 7.34 7.33 7.33 7.32 7.32 7.30
4.49 4.45 4.41 4.37
2.88 2.86 2.85 2.83 2.82 2.81 2.80 2.80 2.78 2.76 2.18 1.74 1.72 1.70 1.69 1.67 1.66 1.64 1.62 1.48 1.46 1.45 1.44 1.43 1.41 1.39 1.38 1.30 1.28 1.25 1.22 1.20 1.19 1.17
^{1}H NMR (400 MHz, DMSO-d_6)
O
OH
O
O
O
O
CH3
O
2d
5.08
2.14
1.10
3.00
2.09
4.24
2.12
7.5 7.0 6.5 6.0 5.5 5.0 4.5 4.0 3.5 3.0 2.5 2.0 1.5
f1 (ppm)
5500000 5000000 4500000 4000000 3500000 3000000 2500000 2000000 1500000 1000000 500000 0

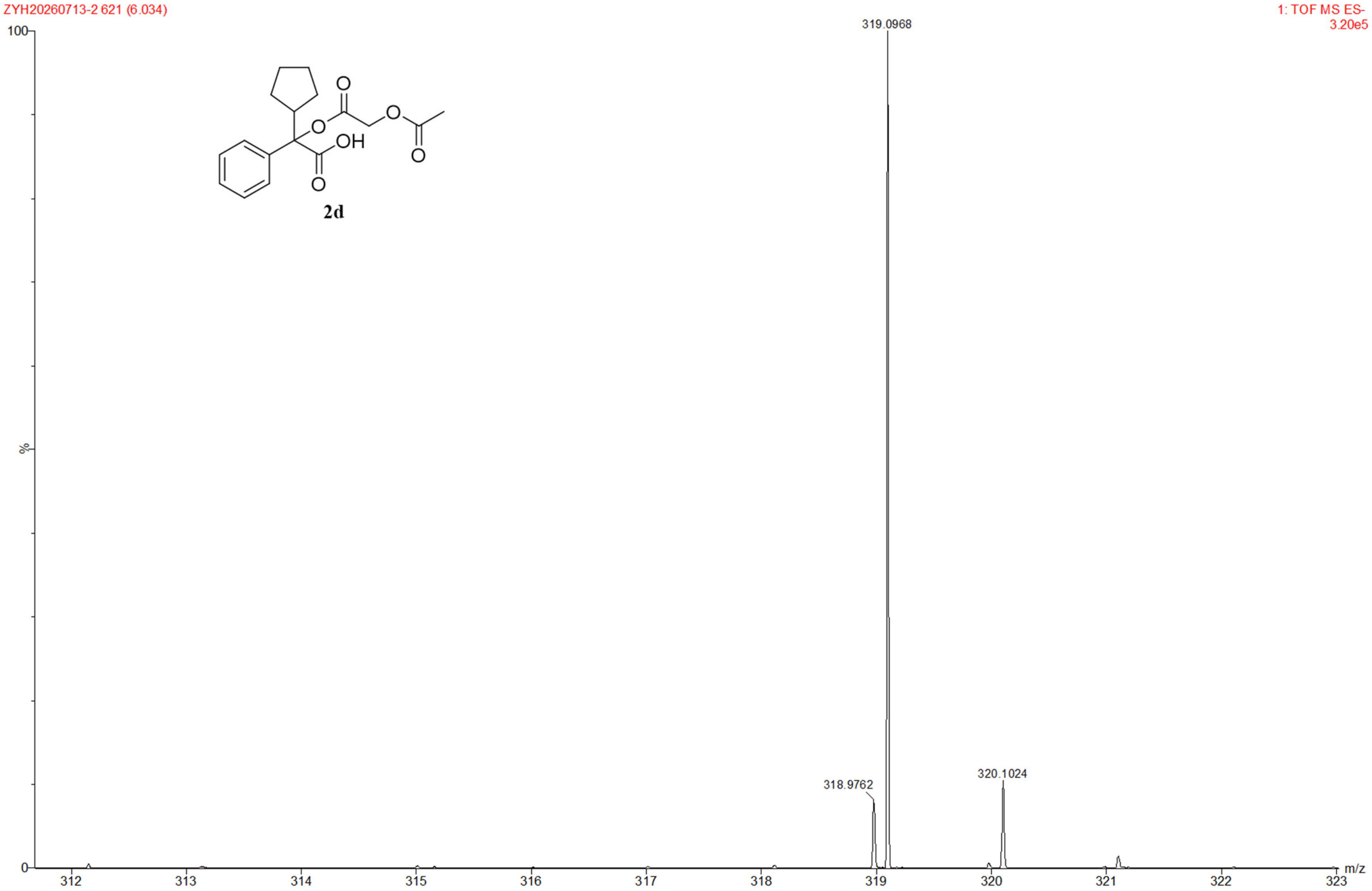
ZYH20260713-2 621 (6.034)
1: TOF MS ES-
3.20e5
2d
319.0968
318.9762
320.1024
100
%
0
312
313
314
315
316
317
318
319
320
321
322
323
m/z

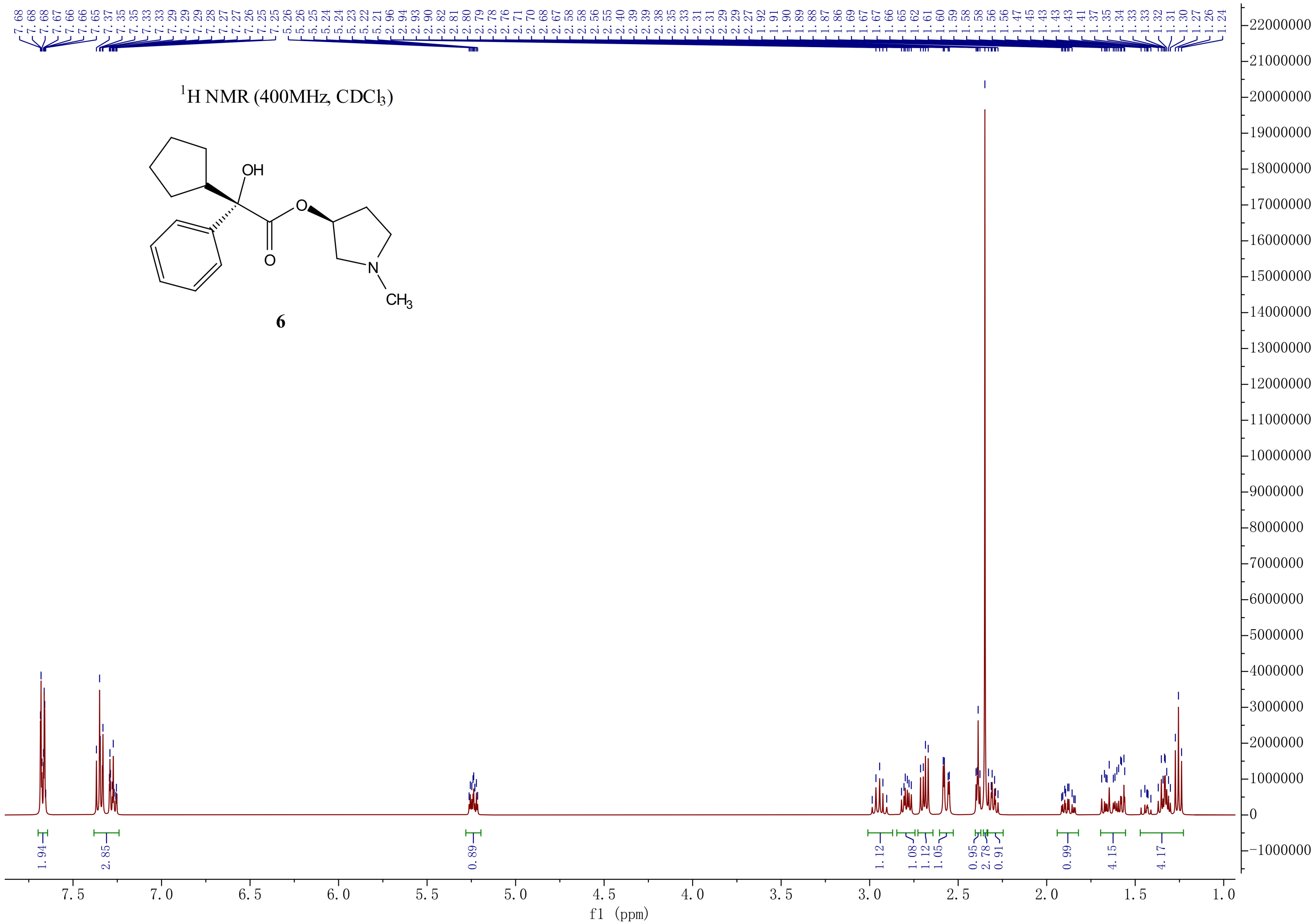
^{1}H NMR (400MHz, CDCl$_3$)
OH
O
O
N
CH$_3$
6
1.94
2.85
0.89
1.12
1.08
1.12
1.05
0.95
2.78
0.91
0.99
4.15
4.17
f1 (ppm)

^{1}H NMR (400MHz, $CDCl_3$)

(S)-counterpart 6

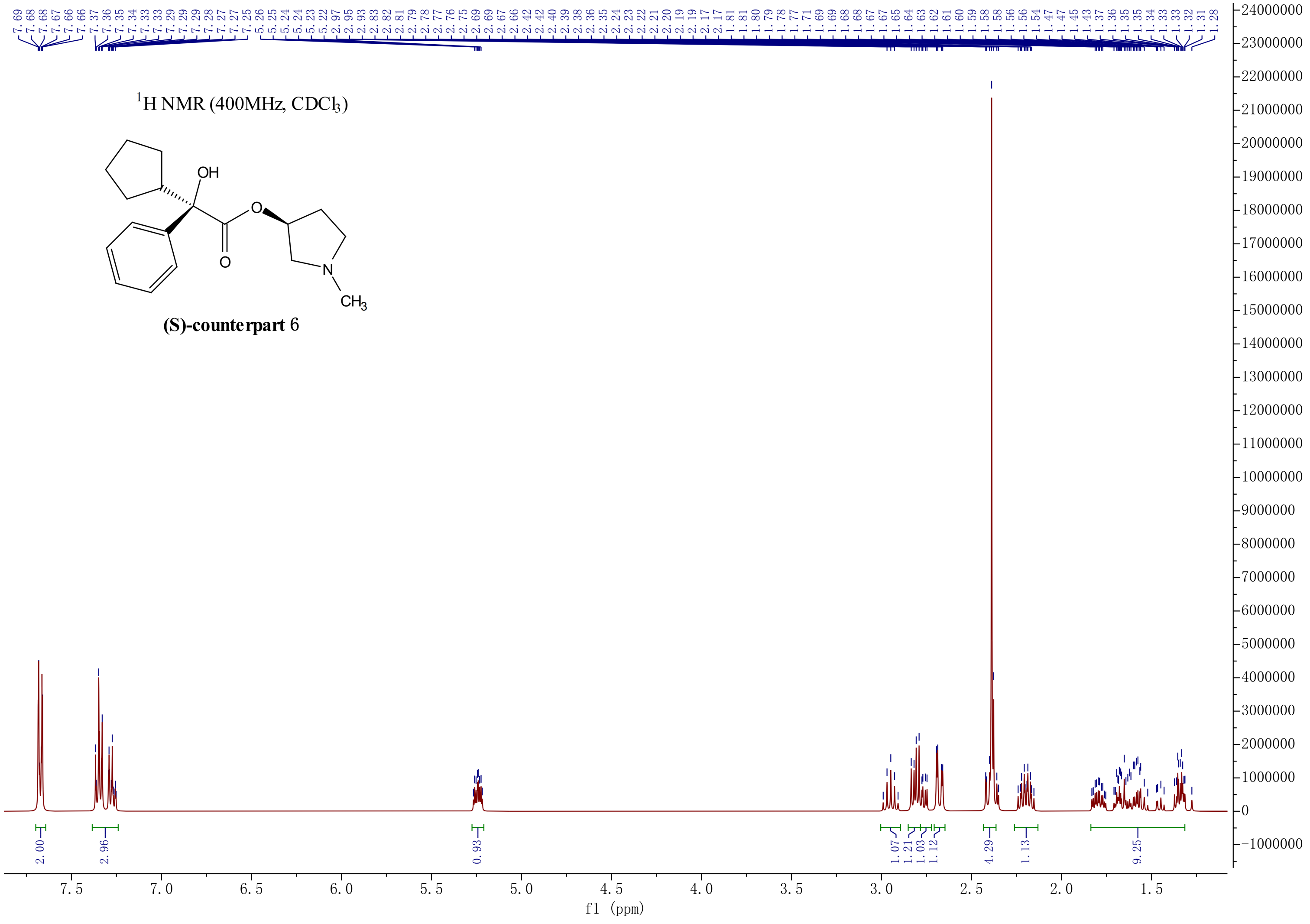

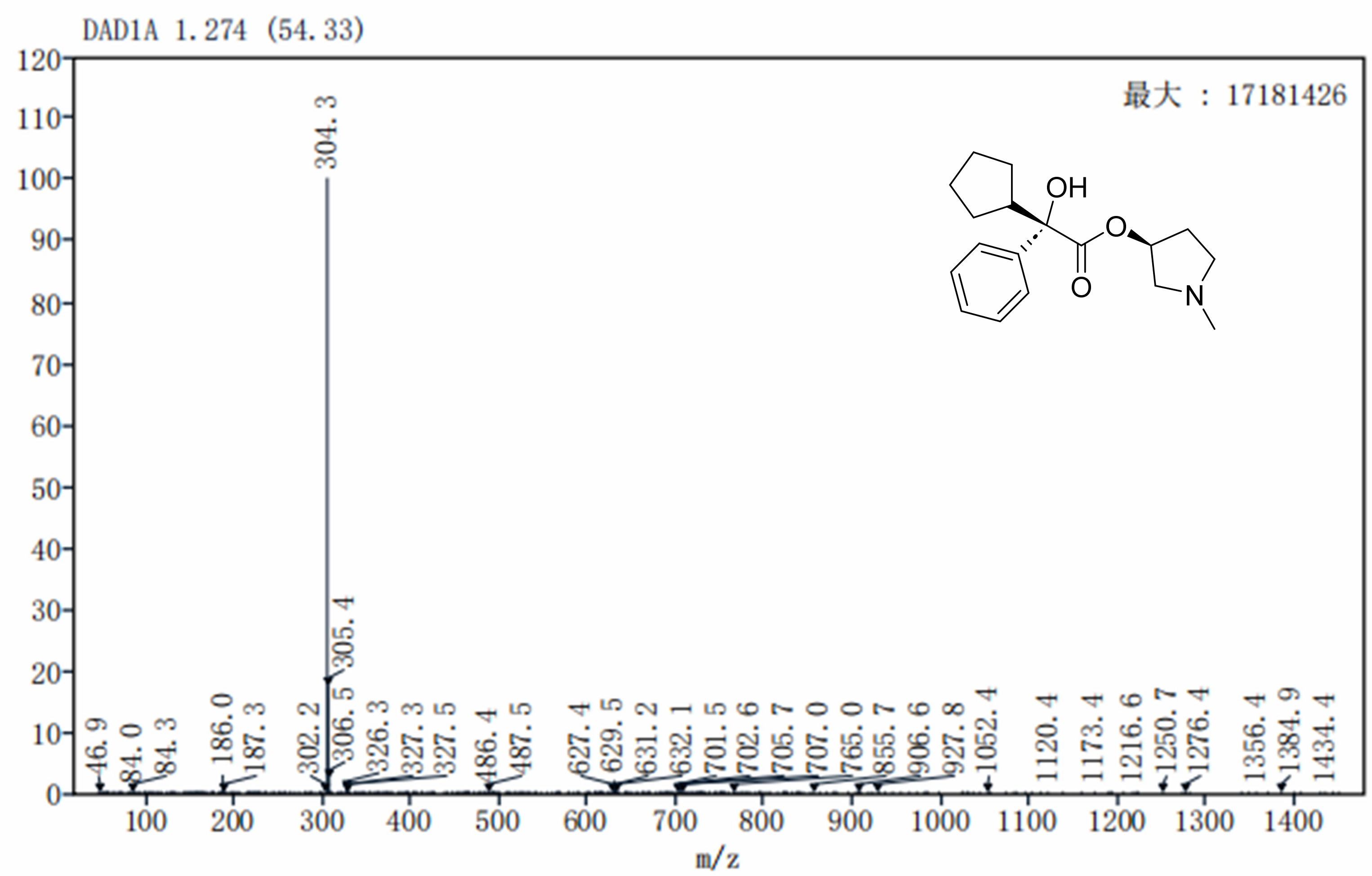

DAD1A 1.274 (54.33)
最大 : 17181426
OH
O
O
N
m/z
304.3
305.4

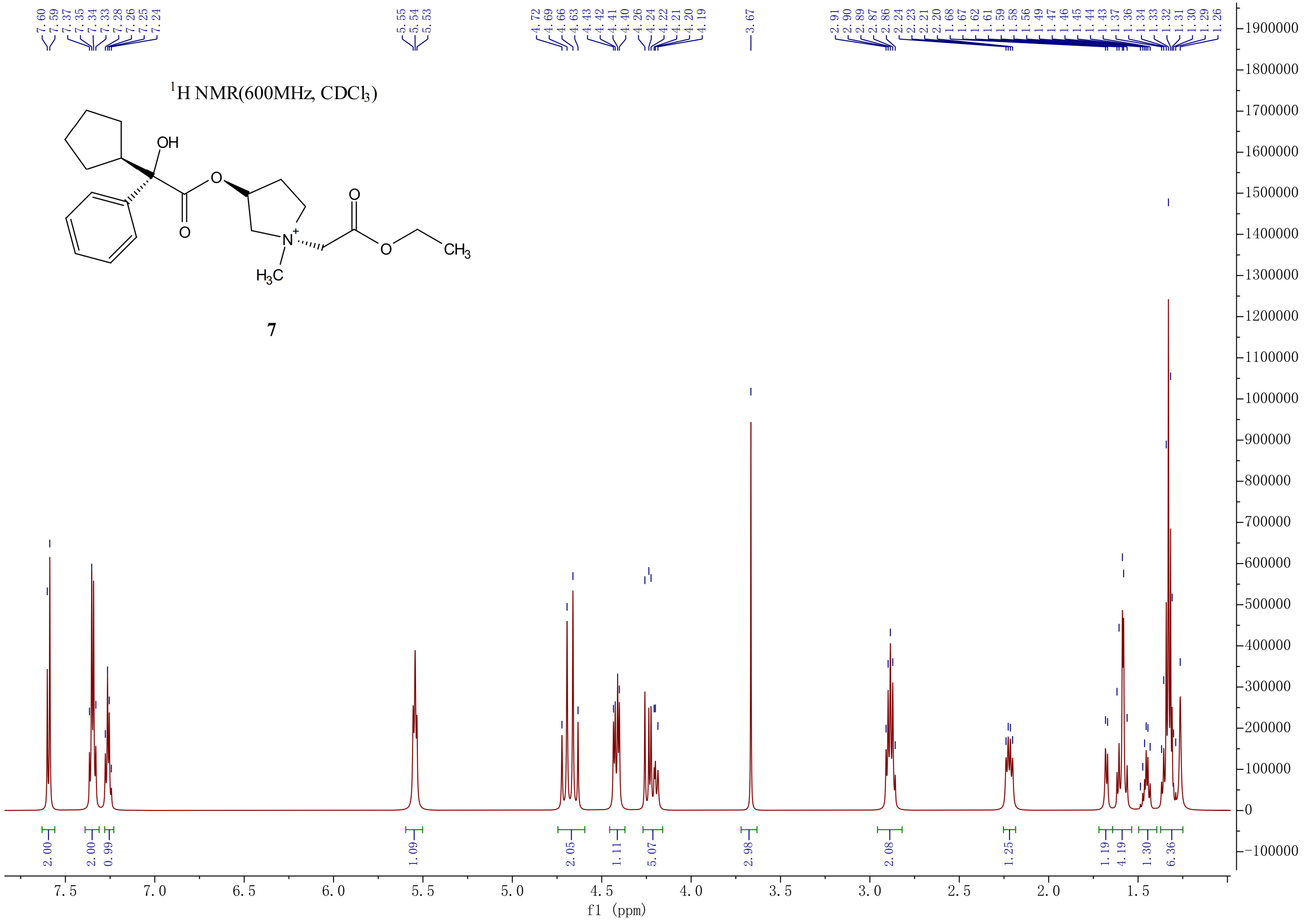
7.60 7.59 7.37 7.35 7.34 7.33 7.28 7.26 7.25 7.24
5.55 5.54 5.53
4.72 4.69 4.66 4.63 4.43 4.42 4.41 4.40 4.26 4.24 4.22 4.21 4.20 4.19
3.67
2.91 2.90 2.89 2.87 2.86 2.24 2.23 2.21 2.20 1.68 1.67 1.62 1.61 1.59 1.58 1.56 1.49 1.47 1.46 1.45 1.44 1.43 1.37 1.36 1.34 1.33 1.32 1.31 1.30 1.29 1.26
1H NMR(600MHz, CDCl3)
OH
O
O
O
N+
O
O
CH3
H3C
7
2.00 2.00 0.99 1.09 2.05 1.11 5.07 2.98 2.08 1.25 1.19 4.19 1.30 6.36
f1 (ppm)

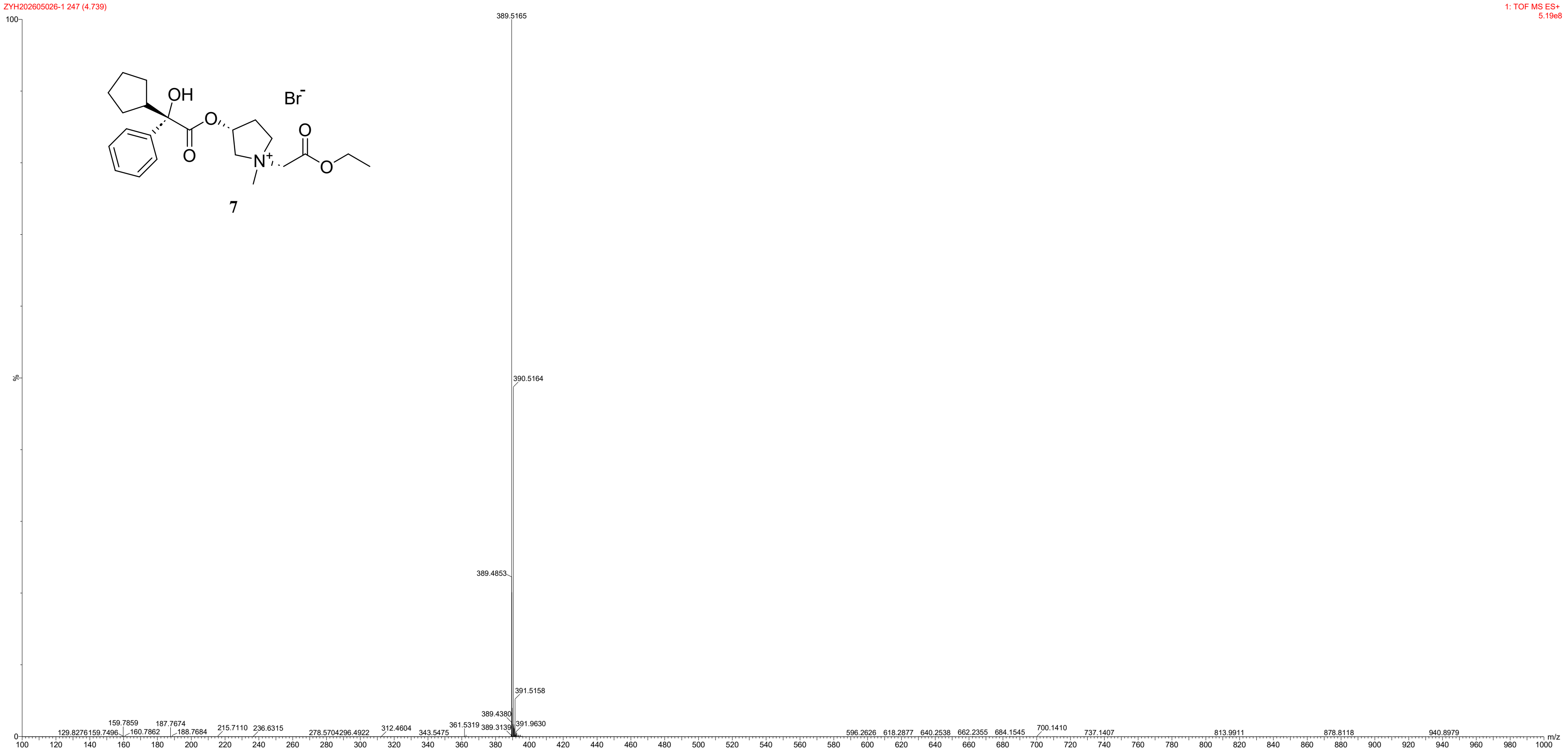
ZYH202605026-1 247 (4.739)
1: TOF MS ES+
5.19e8
389.5165
390.5164
389.4853
391.5158
389.4380
389.3139
391.9630
361.5319
343.5475
312.4604
278.5704
296.4922
236.6315
215.7110
187.7674
188.7684
159.7859
160.7862
129.8276
159.7496
596.2626
618.2877
640.2538
662.2355
684.1545
700.1410
737.1407
813.9911
878.8118
940.8979
m/z
%
OH
O
Br-
N+
7

Representative chiral HPLC chromatogram of (R)- and (S)-CPMA.

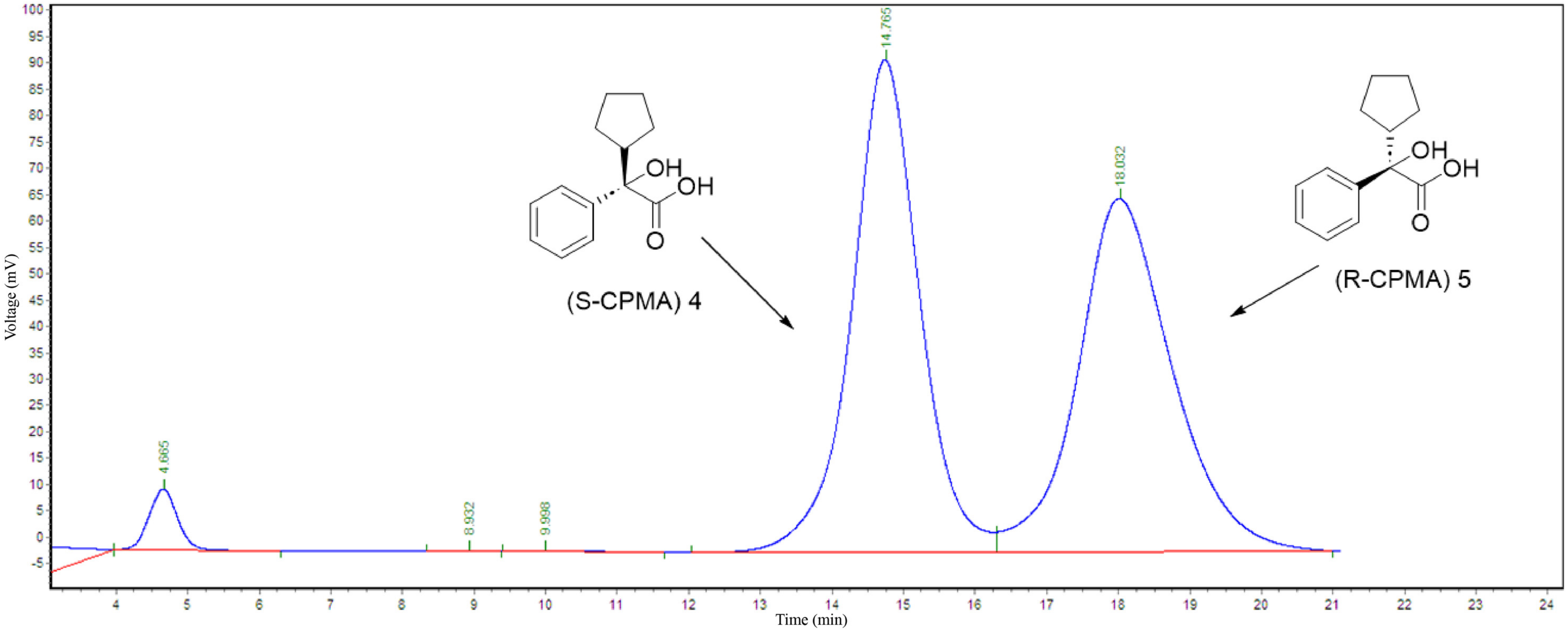

Chiral HPLC chromatogram of (R)-CPMA after enzymatic resolution and deprotection.

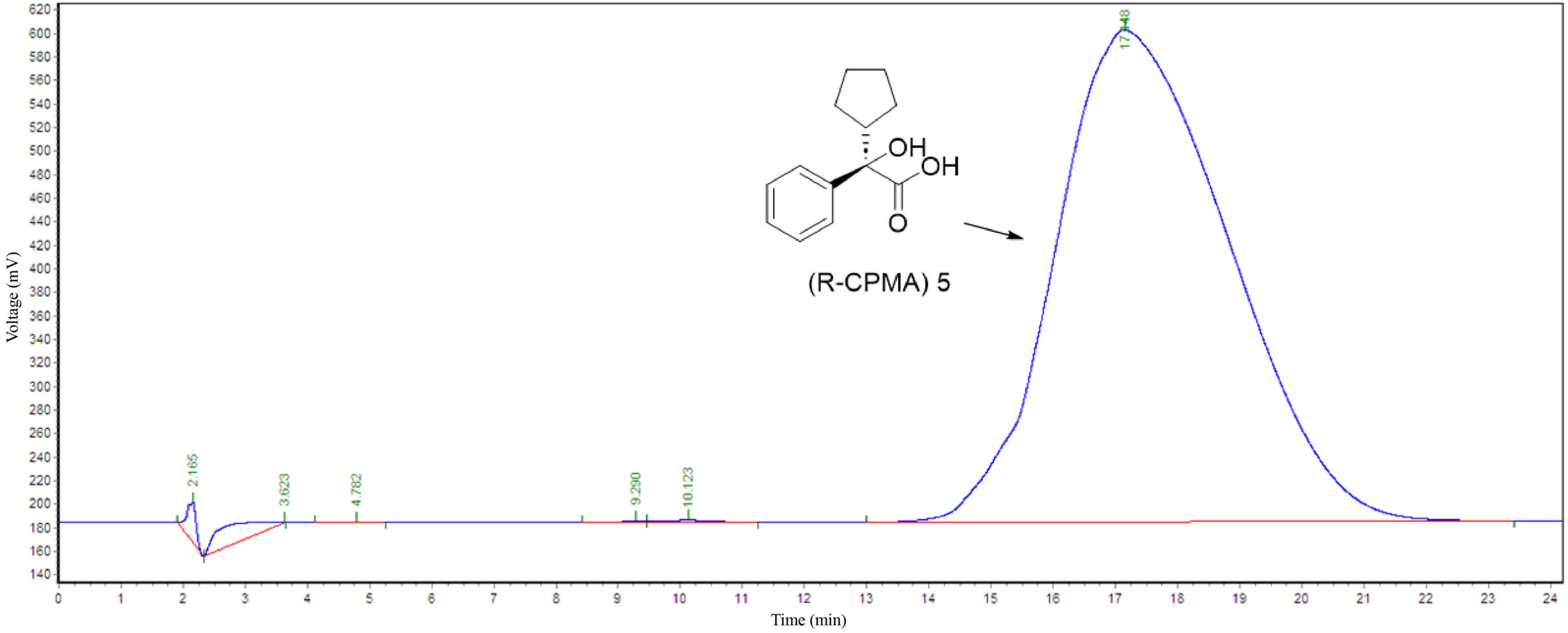

Chiral HPLC chromatogram of (S)-CPMA after enzymatic resolution and deprotection.

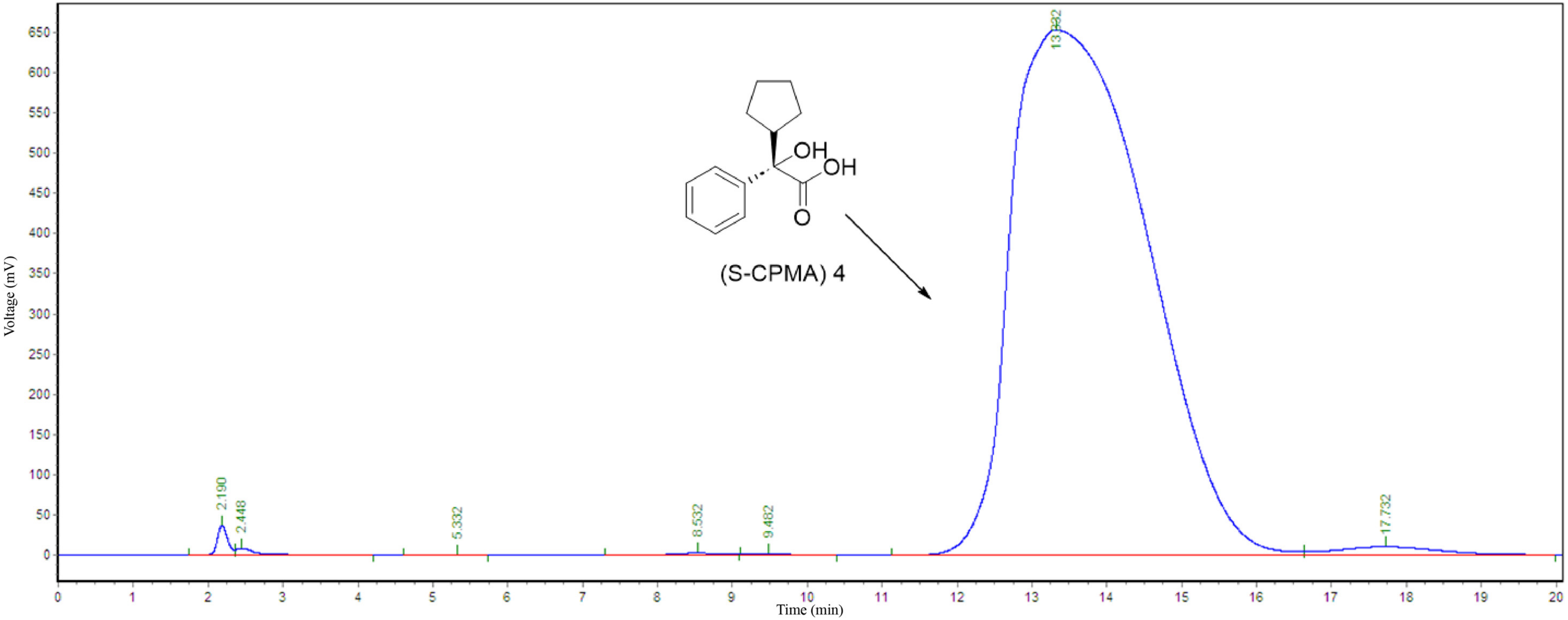

Supplement: Supplementary file 1 [file molecules-31-03290-s001.zip › molecules-4547165-supplementary.pdf]
